# Structured Case-Based Ethics Discussion for Trainees and Faculty on Dermatopathology

**DOI:** 10.15766/mep_2374-8265.11314

**Published:** 2023-05-16

**Authors:** Nathanael C. Jensen, Margaret Cocks, Ben J. Brintz, Benjamin Stoff, Sarah D. Cipriano

**Affiliations:** 1 Fourth-Year Medical Student, University of Utah School of Medicine; 2 Assistant Professor, Department of Dermatology, University of Utah School of Medicine; 3 Assistant Professor, Department of Internal Medicine, University of Utah School of Medicine; 4 Associate Professor, Department of Dermatology, Emory University School of Medicine; 5 Vice Chair of Education, Department of Dermatology, University of Utah School of Medicine

**Keywords:** Case-Based Learning, Dermatoethics, Dermatology, Dermatopathology, Ethics/Bioethics, Professionalism

## Abstract

**Introduction:**

Ethical and professional dilemmas are part of the day-to-day practice of medicine, including within dermatopathology (e.g., ethical implications of self-referring skin biopsies for pathology interpretation). There is a need for teaching aids that dermatology educators can easily access to help provide ethics education.

**Methods:**

We held an hour-long, faculty-facilitated, interactive, virtual discussion about ethical issues in dermatopathology. The session followed a structured, case-based format. We administered anonymous online feedback surveys after the session and used the Wilcoxon signed rank test to compare participants’ before and after responses.

**Results:**

Seventy-two individuals from two academic institutions participated in the session. We collected 35 total responses (49%) from dermatology residents (*n* = 15), dermatology faculty (*n* = 14), medical students (*n* = 2), and other providers and learners (*n* = 4). Feedback was largely positive, with 21 attendees (60%) indicating they learned a few things and 11 (31%) indicating they learned a great deal. Additionally, 32 participants (91%) indicated they would recommend the session to a colleague. Our analysis showed that attendees had a greater self-perceived level of achievement for each of our three objectives after the session.

**Discussion:**

This dermatoethics session is structured so as to be easily shared, deployed, and built on by other institutions. We hope that other institutions will use our materials and results to improve upon the foundation presented here and that this framework will be used by other medical specialties seeking to foster ethics education in their training programs.

## Educational Objectives

By the end of this activity, learners will be able to:

1. Identify ethical and professionalism issues embedded in the day-to-day practice of dermatopathology.
2. Use the four key principles of bioethics to make an ethical argument.
3. Manage ethical conflicts in dermatopathology using appropriate resources.

## Introduction

Dermatopathologists are confronted with a wide range of ethical issues in day-to-day practice, ranging from questionable business tactics to appropriate wording of recommendations on dermatopathology reports. Fortunately, the medical literature contains several resources that help familiarize dermatologists with pertinent ethical dilemmas in modern-day dermatopathology as well as ethical principles to guide decision-making in these scenarios. To this point, though, there have been no widely available teaching aids collating this literature into an organized workshop that dermatology educators can use to help familiarize learners with these issues. For example, a search of *dermatology* in *MedEdPORTAL* revealed 40 educational materials as of June 2022, none of which addresses ethics education. As another example in general pathology, the pathology issue of the *AMA Journal of Ethics* from August 2016 contains many helpful ethical cases and scenarios but does not contain any teaching workshops on dermatopathology.^1^ There is a clear need for additional teaching aids that dermatology educators can easily access to help groups of trainees and faculty recognize ethical issues and learn how to implement ethical principles in clinical and professional practice. Without readily available teaching sessions on dermatology and dermatopathology, this important topic may go unaddressed by dermatology trainees and lead to suboptimal care of dermatology patients.

The Accreditation Council for Graduate Medical Education (ACGME) and the American Board of Dermatology (ABD) continue to emphasize the importance of training in ethics and professionalism in dermatology and dermatopathology. In 2015, a joint initiative between the ACGME, the ABD, and the American Board of Pathology produced both dermatology and dermatopathology milestones, with specific targets for ethics and professionalism.^2,3^ The need for ethics education and training grows because dermatology trainees today face unique ethical challenges (e.g., determining which circumstances are appropriate for teledermatology care, providing elective dermatologic procedures during a pandemic) and must prepare for these in future independent practice.^4–6^ To respond effectively to ethical problems, dermatologists must be able to recognize ethical issues, utilize applicable knowledge and analytical skills, and decide on an appropriate course of action.^7^

To address these needs in dermatology training, an ad hoc subcommittee of the ABD created a model curriculum in ethics and professionalism, which includes pertinent ethics topics and resources for ethics education in dermatology.^8,9^ This model curriculum outlines broad topics in dermatology ethics and includes literature references for each topic; however, it does not provide a readily usable resource that facilitators can deploy to direct dermatoethics sessions.

In an effort to provide a tool that would supplement existing ethics education in dermatology residency programs, we developed a structured discussion-based dermatoethics series for dermatology trainees and faculty. The session presented here addresses ethical issues within dermatopathology. A case-based format was chosen due to the success of this format in delivering ethics education to residents in other specialties.^10^ Additionally, the previously mentioned subcommittee of the ABD recommends a case-based model for content delivery.^8^ Our session encompasses several educational objectives designed to meet ACGME milestones, addresses perceived needs within dermatology departments, and incorporates adapted objectives from Bercovitch and Long's dermatoethics curriculum at Brown Medical School.^4^

## Methods

Our dermatoethics session utilized case- and discussion-based structured teaching to encourage learners to identify and resolve specific ethical conflicts. Because this was the first dermatoethics workshop delivered to the learners at our two participating institutions, we created a short dermatoethics primer presentation to introduce foundational ethical principles to the audience. We presented this primer presentation to all learners prior to beginning the case-based discussion. We gathered content for this primer through institutional access to medical literature and expert consultation, and we prepared the presentation using Microsoft PowerPoint (Appendix A).

Next, we presented a separate case-based PowerPoint (Appendix B) to guide attendees through a series of four case vignettes involving common ethical dilemmas in dermatopathology. We adapted these cases from several sources, including the textbook *Dermatoethics: Contemporary Ethics and Professionalism in Dermatology,*^11^ as well as other published ethical cases identified through literature searches. We evaluated cases based on their ability to illustrate ethical conflicts and principles, generate an engaging discussion, and be viewed by participants as relevant and relatable. We used the following outline for our presentation:

• Educational objectives
• Discussion outline
• Dermatoethics primer (included only in a program's first dermatoethics session)
• Case discussion
  ○ Case details (e.g., clinical vignette)
  ○ Case discussion: multiple-choice questions illustrating various courses of action and open-ended, interactive discussion of ethical considerations and principles
  ○ Supplemental details pertinent to each case (e.g., additional case information, legal information, clinical practice guidelines)
  ○ Further case discussion
  ○ Review of pertinent educational objectives
• References

Due to the COVID-19 pandemic, our facilitated discussion was held virtually. The session took place over an hour with a single large group of participates ranging from medical students to faculty. The facilitator encouraged conversation and helped participants identify the ethical issue(s) pertinent to each case. Additionally, we encouraged attendees to engage in open and honest discussion about the selected topics. All attendees were made aware that their participation would not be used for grading or evaluations of any kind and that the session would not be recorded. Higher-level details regarding delivery of the session are provided here as Appendix C.

Discussions were aimed at advancing the knowledge and professional growth of dermatology residents and faculty but were open to other trainees (medical students, rotating residents, fellows) and nondermatology faculty as well. We informed dermatology residents that their attendance was expected (but not mandatory); attendings were encouraged to join and participate. We held the sessions virtually over Zoom at two institutions: University of Utah and Emory University. We designed these discussions to be informal and encouraged active, respectful participation.

We used anonymous feedback surveys (Appendix D) to evaluate current sessions and improve future series. Each person in attendance received a link to an online survey and was encouraged to fill it out after the meeting. We obtained information about participant satisfaction with both discrete and free-response questions. We used this information for session feedback and longitudinal improvements to the sessions. We evaluated discrete questions using standardized measures and analyzed free responses individually. We noted general themes and then categorized responses and counted according to these themes. To evaluate the effect of the session on our three objectives, we asked attendees to assign a self-perceived level of achievement to each objective, with 1 = no achievement, 2 = low achievement, 3 = moderate achievement, and 4 = high achievement. Attendees assigned both a presession level of achievement and a postsession level of achievement. We hypothesized that as a result of participating in the session, participants would report a higher postsession self-perceived level of achievement in the three objectives.

We performed the Wilcoxon signed rank test, a nonparametric test for paired data, using a two-sided alternative, between the presession rating and the postsession rating to assess if session attendees experienced a change in the three objectives. We used a statistical significance threshold of .05 for all statistical tests.

## Results

Seventy-two individuals (attendings, residents, students, and other learners) from two academic institutions participated in the session. We received a total of 35 (49%) survey responses. We collected feedback from dermatology residents (*n* = 15), dermatology faculty (*n* = 14), medical students (*n* = 2), and other providers and learners (*n* = 4). Feedback was largely positive, with 60% (*n* = 21) of attendees indicating they learned a few things and 31% (*n* = 11) indicating they learned a great deal. Additionally, 91% (*n* = 32) said they would recommend the session to a colleague.

Participants’ self-perceived level of achievement in “identifying ethical and professionalism issues embedded in the day-to-day practice of dermatology” increased from a median of 3 (interquartile range [IQR]: 2–3) to 3 (IQR: 3–4), *p* < .001. Participants’ self-perceived level of achievement in “using the four key principles of bioethics to make an ethical argument” increased from a median of 3 (IQR: 2–3) to 3 (IQR: 3–4), *p* < .001. Participants’ self-perceived level of achievement in “managing ethical conflicts in dermatology using appropriate resources” changed from a median of 2 (IQR: 2–3) to 3 (IQR: 3–3), *p* < .001. With *p* values less than .05, there was a statistically significant increase in the median of the participants’ responses from before to after the session for all three objectives. Overall, the majority of the participants’ self-perceived level of achievement either stayed the same or improved from before the session to after the session in each of the three categories.

We asked attendees, “What changes will you incorporate into your future practice as a result of the knowledge/perspectives acquired during this activity?” Eight attendees responded that they would work to improve their communication with dermatopathologists. Their comments included “Be more specific on pathology requests” and “I will attempt more often to contact the dermatopathologist involved if I am concerned about their treatment suggestions.” Nine attendees addressed increased awareness of cost to underinsured patients. For example, one respondent replied, “Be consistently aware of costs and their necessity as they relate to work up of various problems,” and another participant stated, “Be cognizant of communication with patients who are self-pay on cost and decision-making.” Other similar comments included “Consider the downstream implications of biopsies in the underinsured going forward” and “Understanding the cost of dermatopathology and ethical issues surrounding it in the future.”

We also asked participants about their favorite aspect of the session. The majority of attendees liked the structured discussion format of the session using a case-based format. Some attendees also commented that they enjoyed having dermatopathologists present in the session who could speak to their personal experiences with the ethical challenges presented. Several participants liked the discussion on direct versus client billing and the legal and ethical implications of this practice as presented in Appendix B. Attendees’ least favorite parts of the session included the virtual format due to the COVID-19 pandemic, absence of a practical solution to some of the ethical scenarios, struggling to find application to daily practice in general dermatology, and lack of time to cover more scenarios due to the length of discussion on each case.

To improve the session in the future, attendees suggested that there be more cases presented at a faster pace and a longer session to address more content. There were also suggestions to increase audience interaction, change to an in-person format, and include personal examples with both positive and negative results. Some topics suggested for future discussions included ethical issues around social media, difficult patients, product promotion, consultative dermatology, discharging patients from clinic, insurance issues, cosmetic procedures during a pandemic, and procedures for those of advanced age.

## Discussion

Our broad goals when designing and implementing this dermatoethics session were twofold: first, to assist dermatologists in training and in practice to build an ethical foundation that would guide a lifetime of practice; second, to design and structure sessions that could easily be shared, deployed, and built on by other institutions. Interestingly, some of the suggestions for future discussion topics identified by our departments are already items recommended by the ABD.^8^

Regarding our findings, participants clearly benefitted from the session and perceived that they would have more confidence in addressing ethical issues around dermatopathology in practice. This indicates that case-based ethical discussions, such as this one, can be an effective method of helping medical learners become more confident in managing ethical situations. The development of this session brought our attention to the fact that there are many high-quality articles in the literature addressing ethical issues in dermatology. However, very few of these had been collated into one learning resource. While implementing this session, some challenges we faced included audience engagement over a virtual platform, sufficient time to define necessary terms and laws for the audience, familiarity with fundamental ethical principles, and lack of personal examples with some of the ethical scenarios prevented.

These challenges provided some important suggestions for those implementing this session in the future. First, for programs presenting an ethics workshop for the first time, we recommend presenting the provided dermatoethics primer presentation (Appendix A) prior to the case-based discussion (Appendix B) in order to introduce foundational ethical principles to the audience. Second, ensure that one or more dermatopathologists or pathologists take part in the session. They can participate as either a facilitator or an audience member. This will introduce more personal experience and familiarity with these issues from a practical standpoint and help participants gain an appreciation for these issues. Third, if circumstances permit, we recommend holding this session in person to maximize audience engagement. Finally, should institutions wish to administer a survey to evaluate participants’ opinion of the session, we suggest designating approximately 5 minutes at the end of the session for participants to complete the survey. This will likely improve response rates.

Limitations of this project include the lack of a control group for comparison. All survey data were self-reported by the participants immediately after the session, limiting validity assessment of durable effects of the session and accuracy of responses. The self-report format and survey timing also introduced several forms of bias, such as recency bias and social desirability bias. Additionally, to determine if the objectives had been met, we asked participants to report their self-perceived level of achievement with each objective on a level of 1–4. Our survey did not define the difference between low, moderate, and high achievement. As such, even though there was a significant increase in attendees’ self-perceived achievement in all three objectives, our results are limited by the participants’ perception of what each level of achievement entails. This means that we could not evaluate whether our objectives were truly achieved, only that participants perceived themselves to be able to achieve them. Due to the limitations on physical gathering, the sessions were carried out over videoconferencing platforms, which may have limited the quality of discussion and level of participation. Lastly, about half (49%) of the session attendees filled out the survey. Thus, our results may exhibit some nonresponse bias. We believe this low response rate may have been a result of the virtual nature of the session. Additionally, we did not provide protected time during the end of the session for participants to complete the online survey. Had the session been held in person, we would likely have been able to improve the response rate by providing each audience member with a physical copy of the survey and asking that they return it on their way out.

Our intention is that the resources provided here can be implemented at other institutions. Additionally, we hope that other medical specialties can use some of our lessons as a framework for their own ethics education and professional development. While not everything will apply to nondermatology specialties, many of the ethical dilemmas we discussed are ubiquitous throughout medicine and can be used as a starting point for ethics discussions and trainings. We are hopeful that this session will help prepare learners to work through the ethical challenges they face in their day-to-day practice.^5^ We aim to present data from our other dermatoethics sessions developed through this collaboration in the near future.

## Appendices


Dermatoethics Primer.pptxEthics in Dermatopathology.pptxFacilitators Guide.docxFeedback Survey.docx
*All appendices are peer reviewed as integral parts of the Original Publication.*
