# Supplementary material for: Structured Case-Based Ethics Discussion for Trainees and Faculty on Dermatopathology
Source: MedEdPORTAL. 2023 May 16;19:11314. doi: 10.15766/mep_2374-8265.11314 (PMC10185701; doi:10.15766/mep_2374-8265.11314)
Supplement: Supplementary file 1 — Dermatoethics Primer.pptxEthics in Dermatopathology.pptxFacilitators Guide.docxFeedback Survey.docx [file mep_2374-8265.11314-s001.zip › A. Dermatoethics Primer.pptx]

## Slide 1
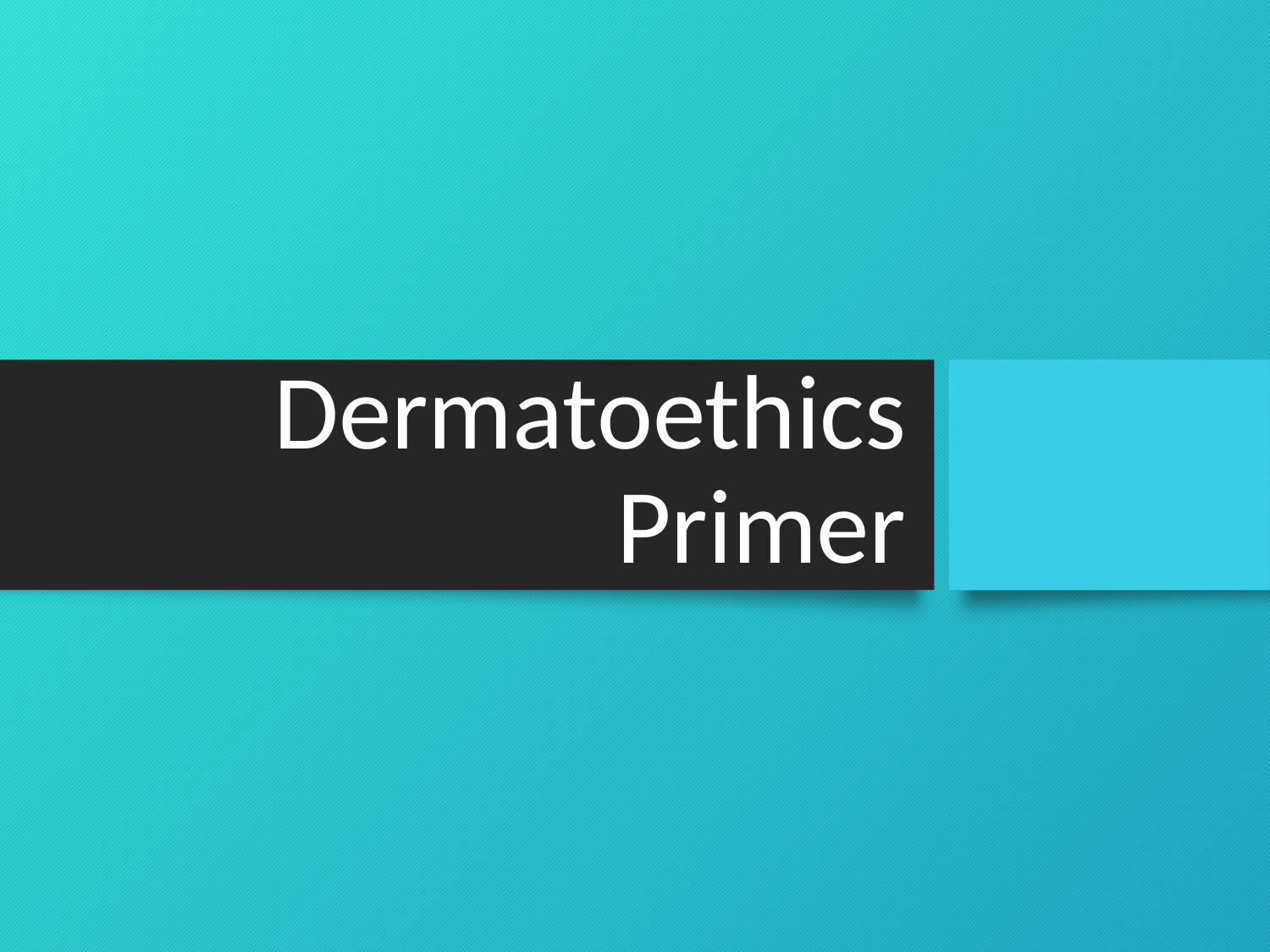

# Dermatoethics Primer

## Slide 2
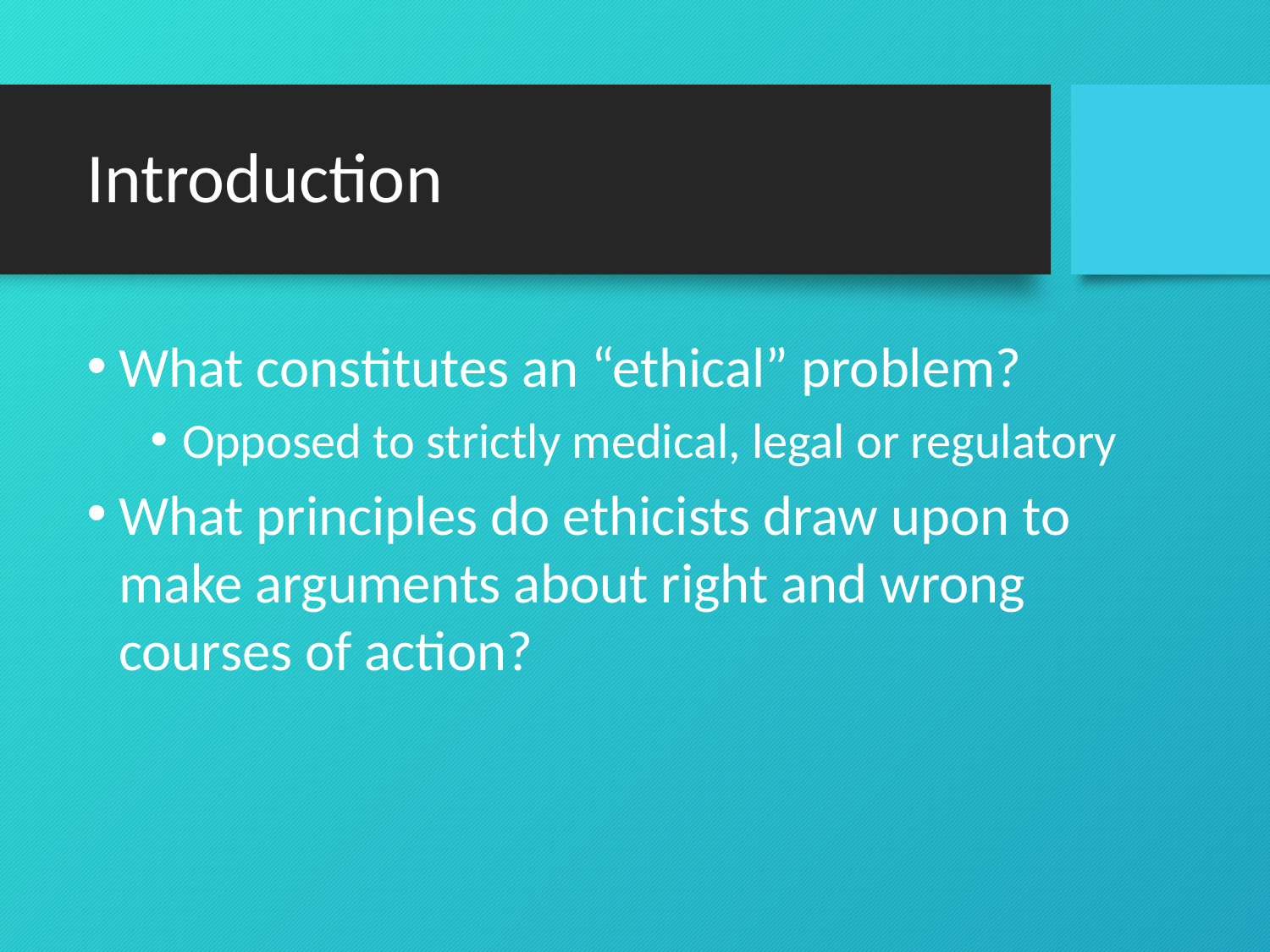

# Introduction
What constitutes an “ethical” problem?
Opposed to strictly medical, legal or regulatory
What principles do ethicists draw upon to make arguments about right and wrong courses of action?

## Slide 3
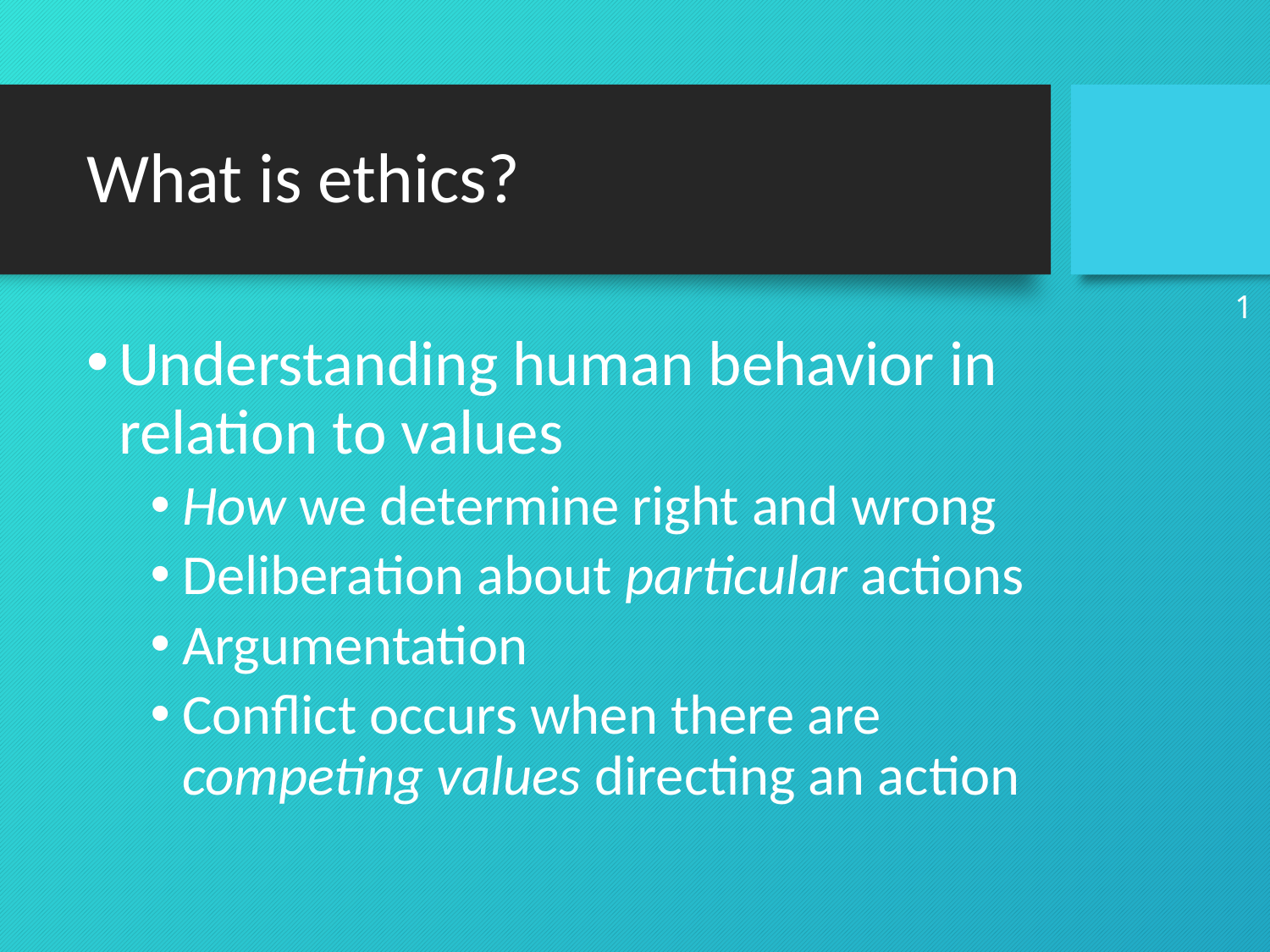

# What is ethics?
1
Understanding human behavior in relation to values
How we determine right and wrong
Deliberation about particular actions
Argumentation
Conflict occurs when there are competing values directing an action

## Slide 4
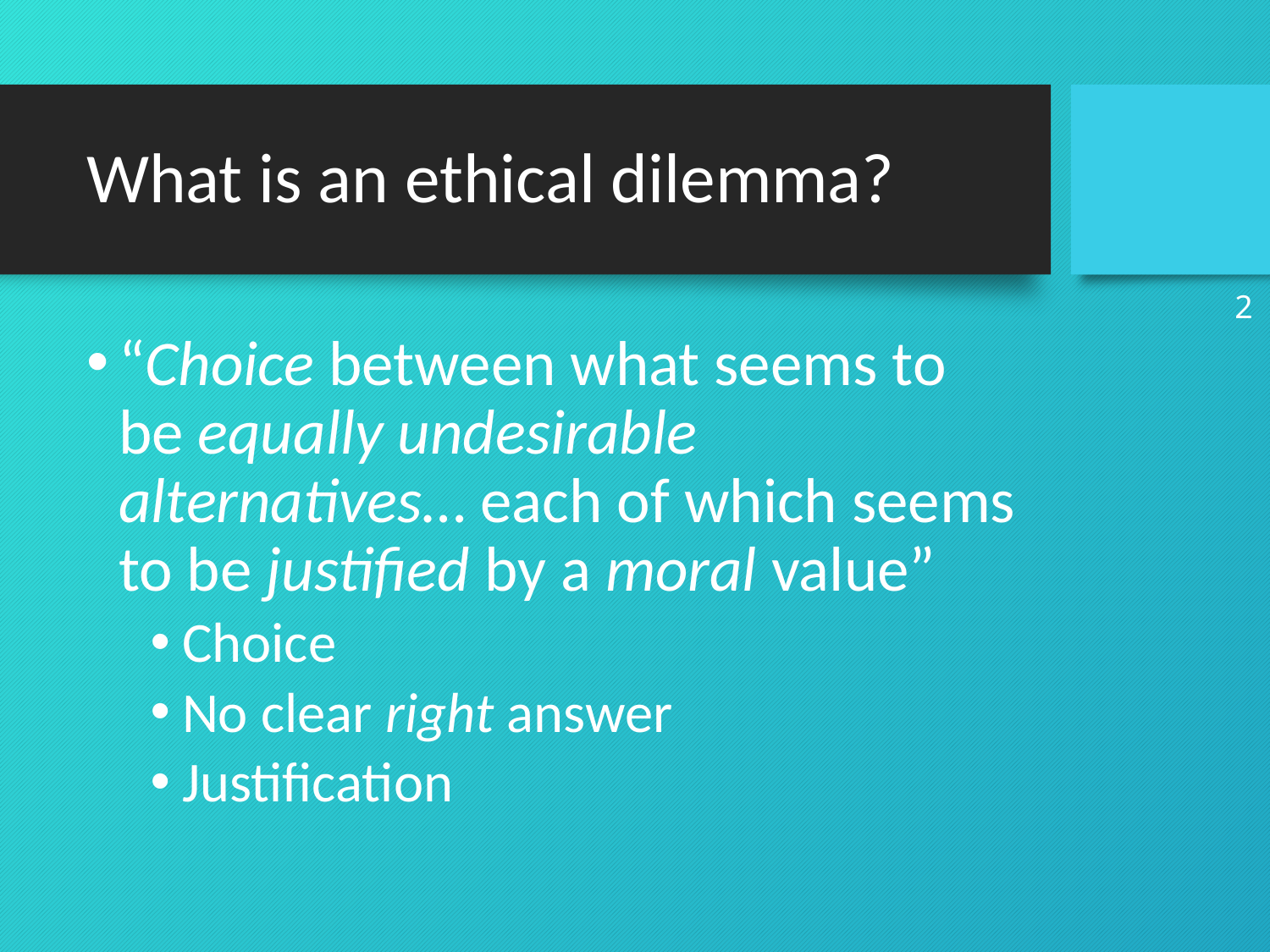

# What is an ethical dilemma?
2
“Choice between what seems to be equally undesirable alternatives… each of which seems to be justified by a moral value”
Choice
No clear right answer
Justification

## Slide 5
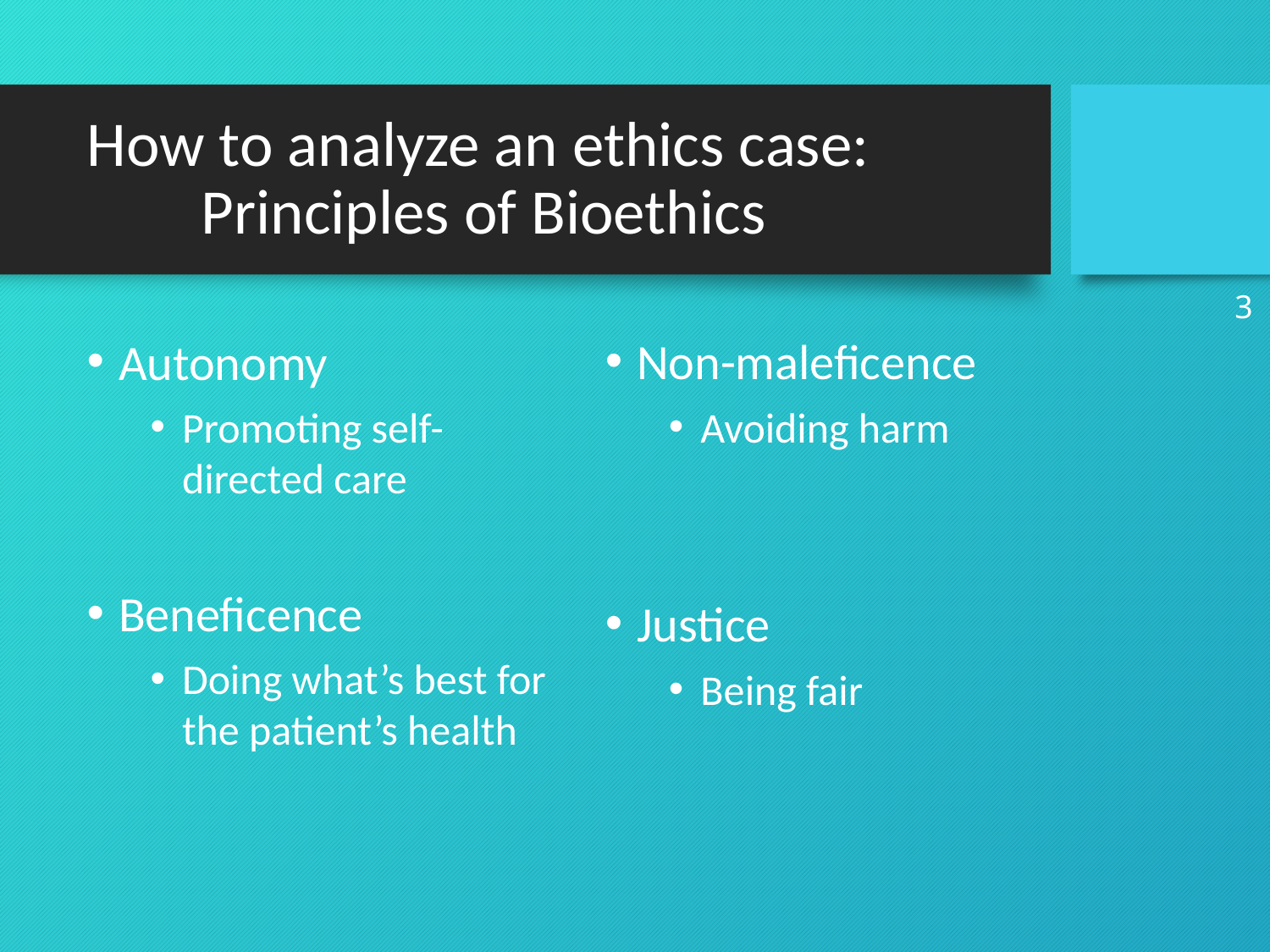

# How to analyze an ethics case: Principles of Bioethics
3
Autonomy
Promoting self-directed care
Beneficence
Doing what’s best for the patient’s health
Non-maleficence
Avoiding harm
Justice
Being fair

## Slide 6
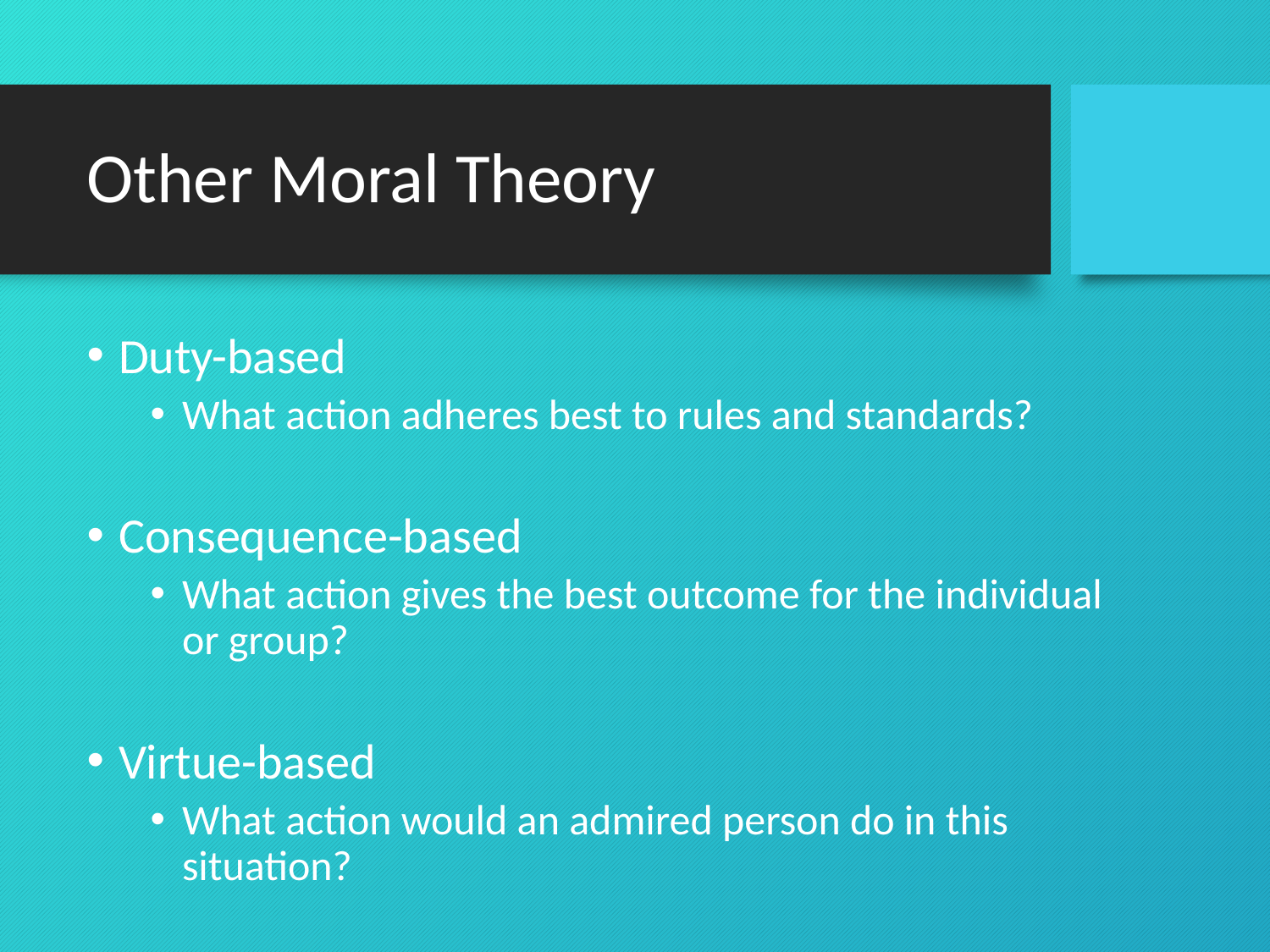

# Other Moral Theory
Duty-based
What action adheres best to rules and standards?
Consequence-based
What action gives the best outcome for the individual or group?
Virtue-based
What action would an admired person do in this situation?

## Slide 7
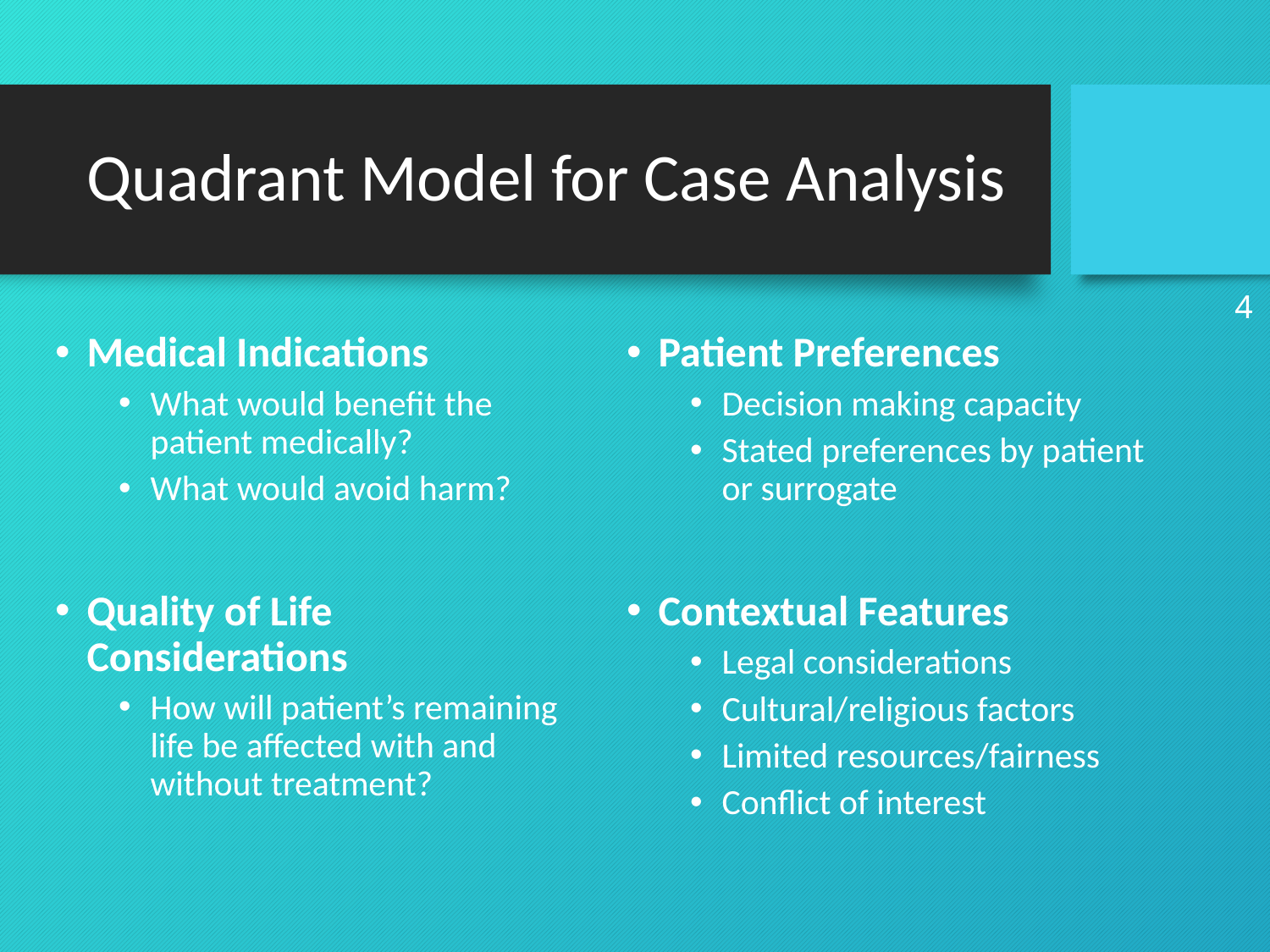

# Quadrant Model for Case Analysis
4
Medical Indications
What would benefit the patient medically?
What would avoid harm?
Quality of Life Considerations
How will patient’s remaining life be affected with and without treatment?
Patient Preferences
Decision making capacity
Stated preferences by patient or surrogate
Contextual Features
Legal considerations
Cultural/religious factors
Limited resources/fairness
Conflict of interest

## Slide 8
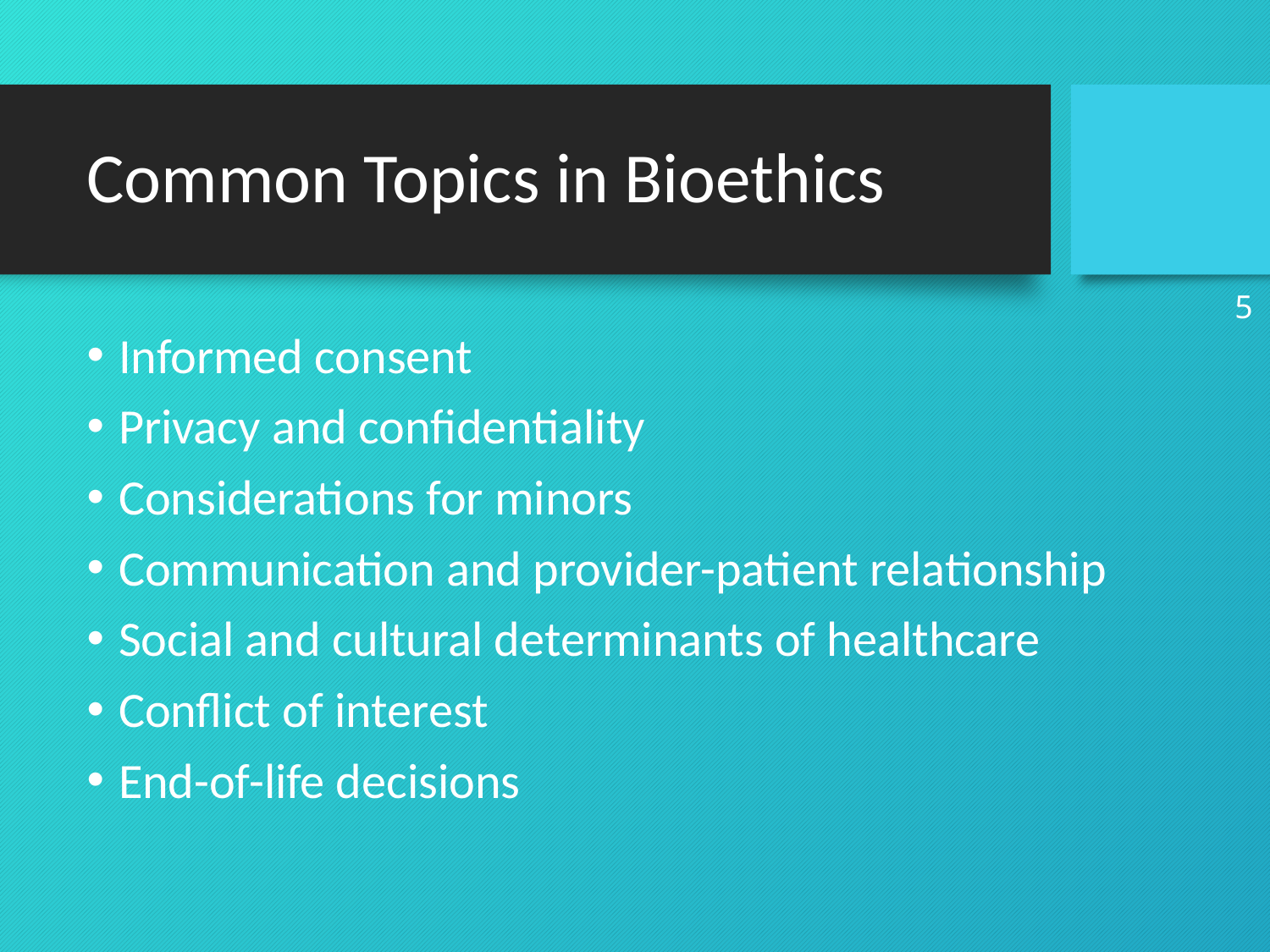

# Common Topics in Bioethics
5
Informed consent
Privacy and confidentiality
Considerations for minors
Communication and provider-patient relationship
Social and cultural determinants of healthcare
Conflict of interest
End-of-life decisions

## Slide 9
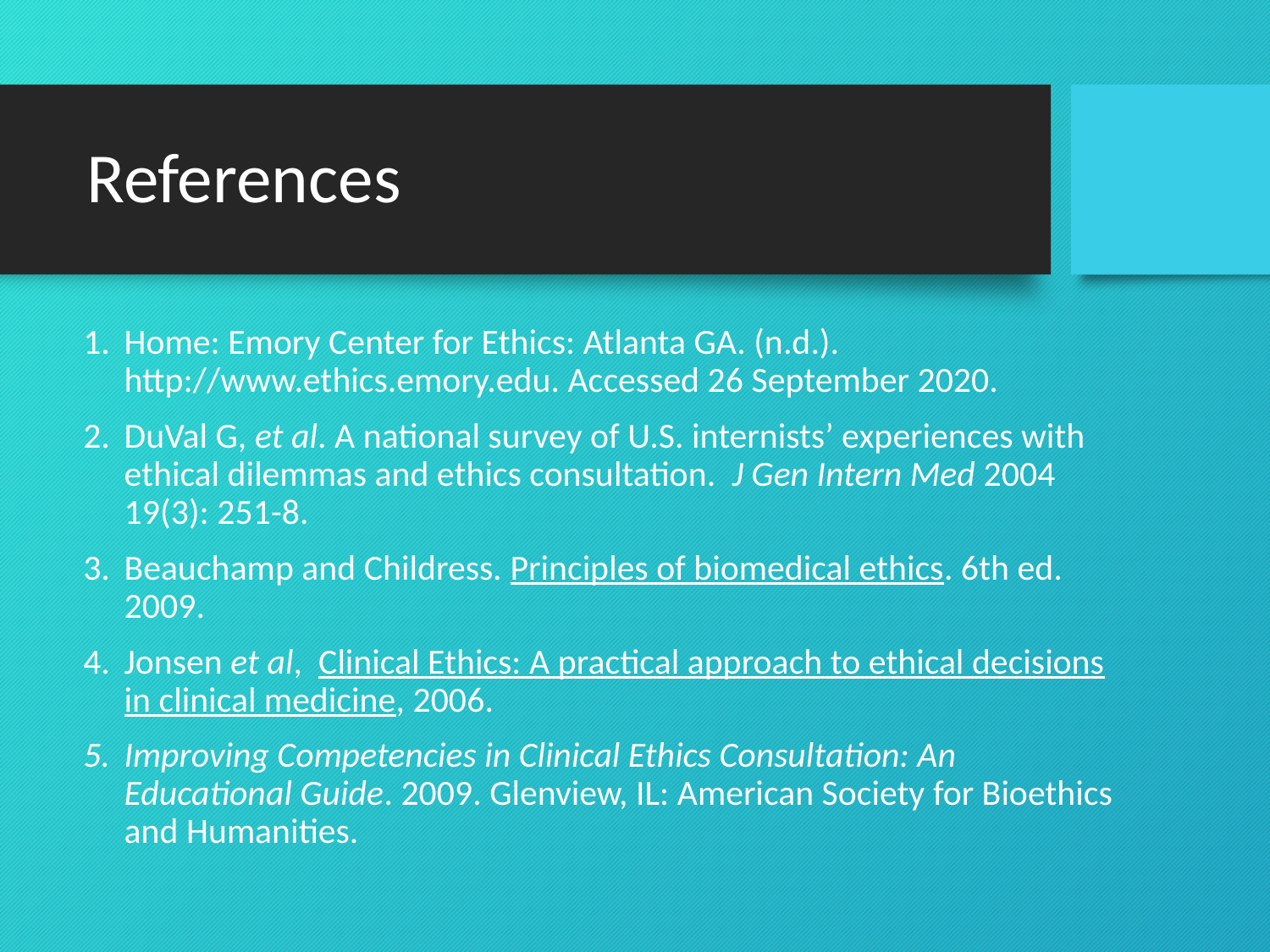

# References
Home: Emory Center for Ethics: Atlanta GA. (n.d.). http://www.ethics.emory.edu. Accessed 26 September 2020.
DuVal G, et al. A national survey of U.S. internists’ experiences with ethical dilemmas and ethics consultation. J Gen Intern Med 2004 19(3): 251-8.
Beauchamp and Childress. Principles of biomedical ethics. 6th ed. 2009.
Jonsen et al, Clinical Ethics: A practical approach to ethical decisions in clinical medicine, 2006.
Improving Competencies in Clinical Ethics Consultation: An Educational Guide. 2009. Glenview, IL: American Society for Bioethics and Humanities.
