# Supplementary material for: Structured Case-Based Ethics Discussion for Trainees and Faculty on Dermatopathology
Source: MedEdPORTAL. 2023 May 16;19:11314. doi: 10.15766/mep_2374-8265.11314 (PMC10185701; doi:10.15766/mep_2374-8265.11314)
Supplement: Supplementary file 1 — Dermatoethics Primer.pptxEthics in Dermatopathology.pptxFacilitators Guide.docxFeedback Survey.docx [file mep_2374-8265.11314-s001.zip › B. Ethics in Dermatopathology.pptx]

## Slide 1
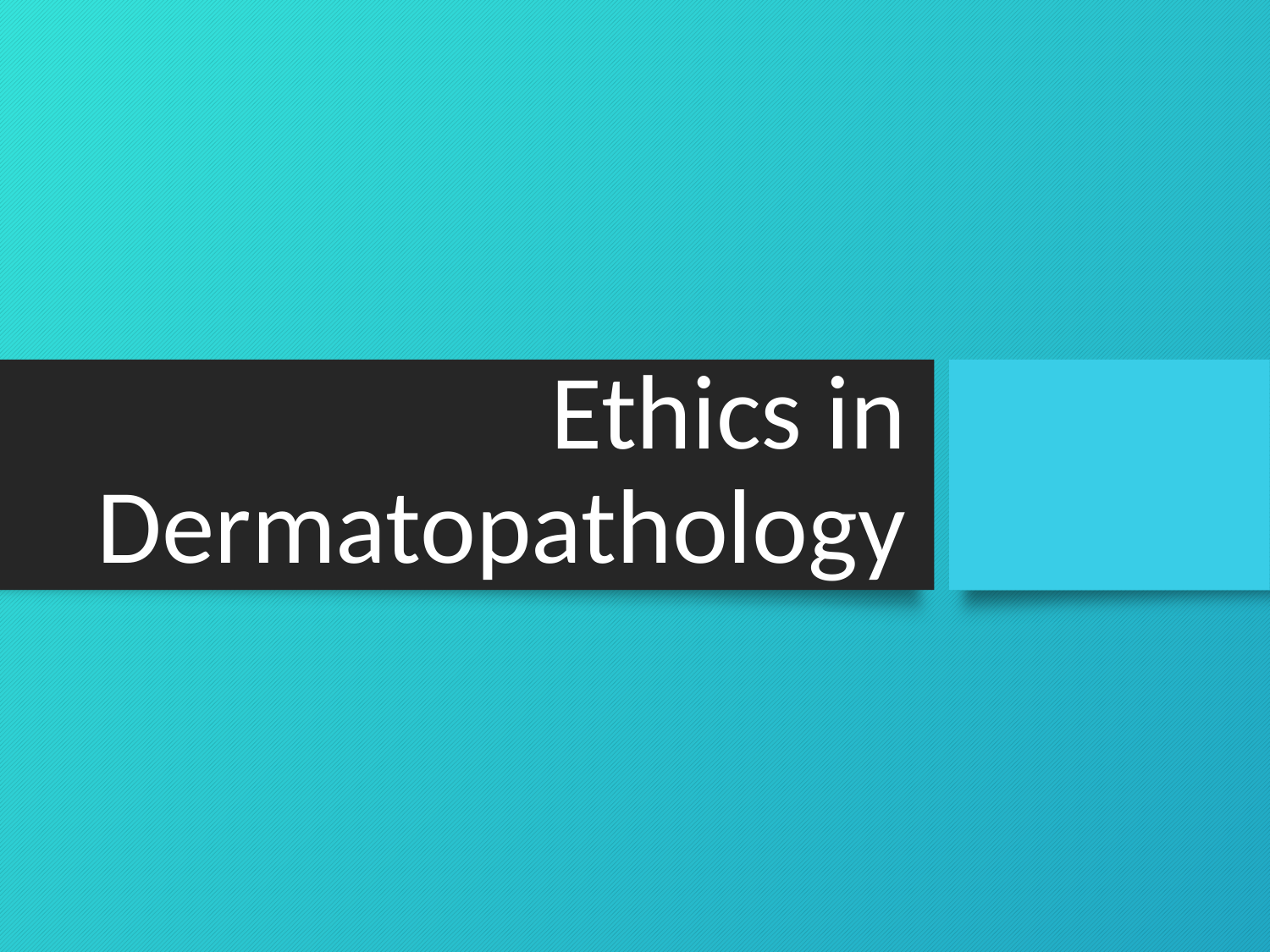

# Ethics in Dermatopathology

## Slide 2
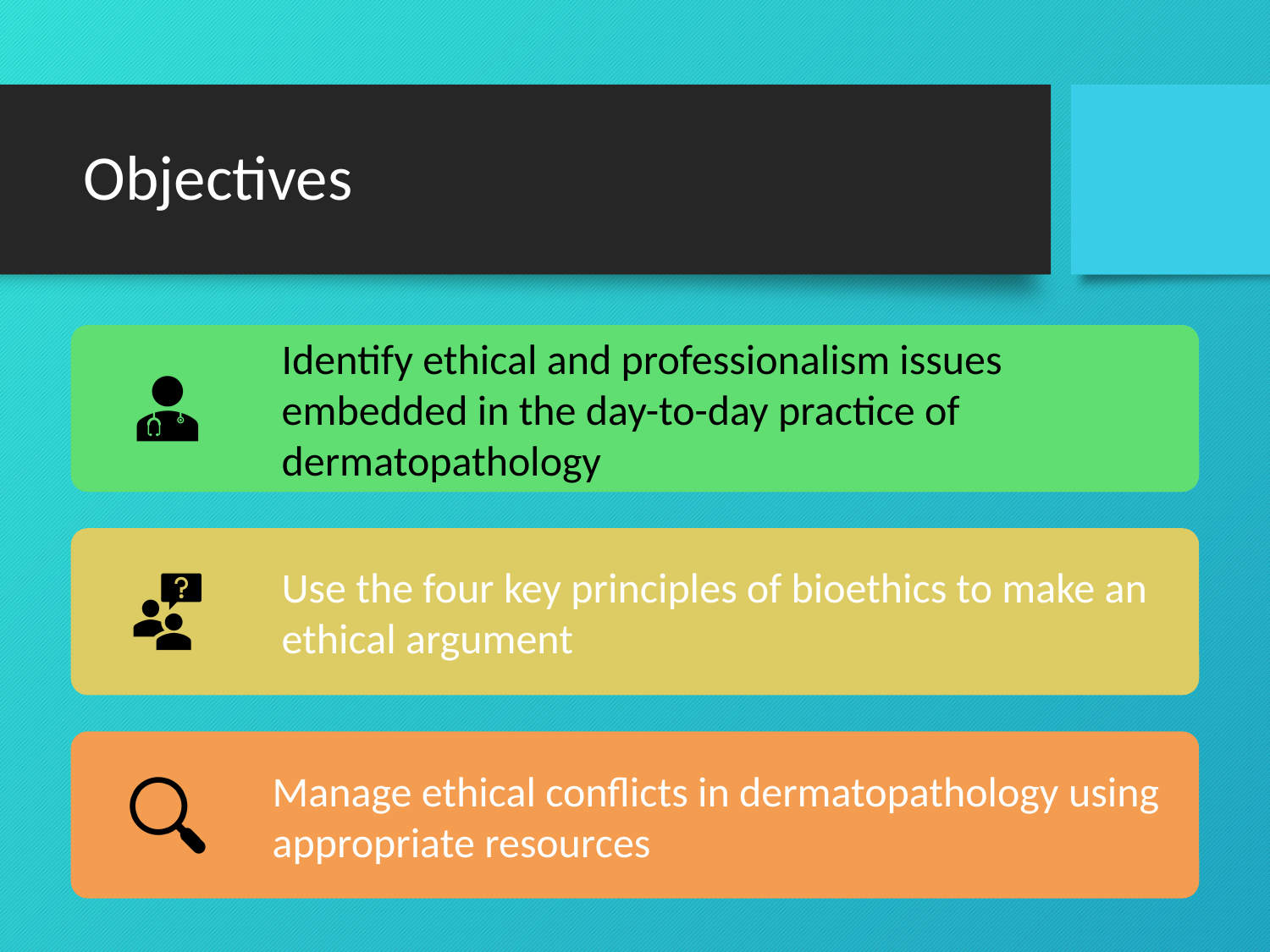

# Objectives

## Slide 3
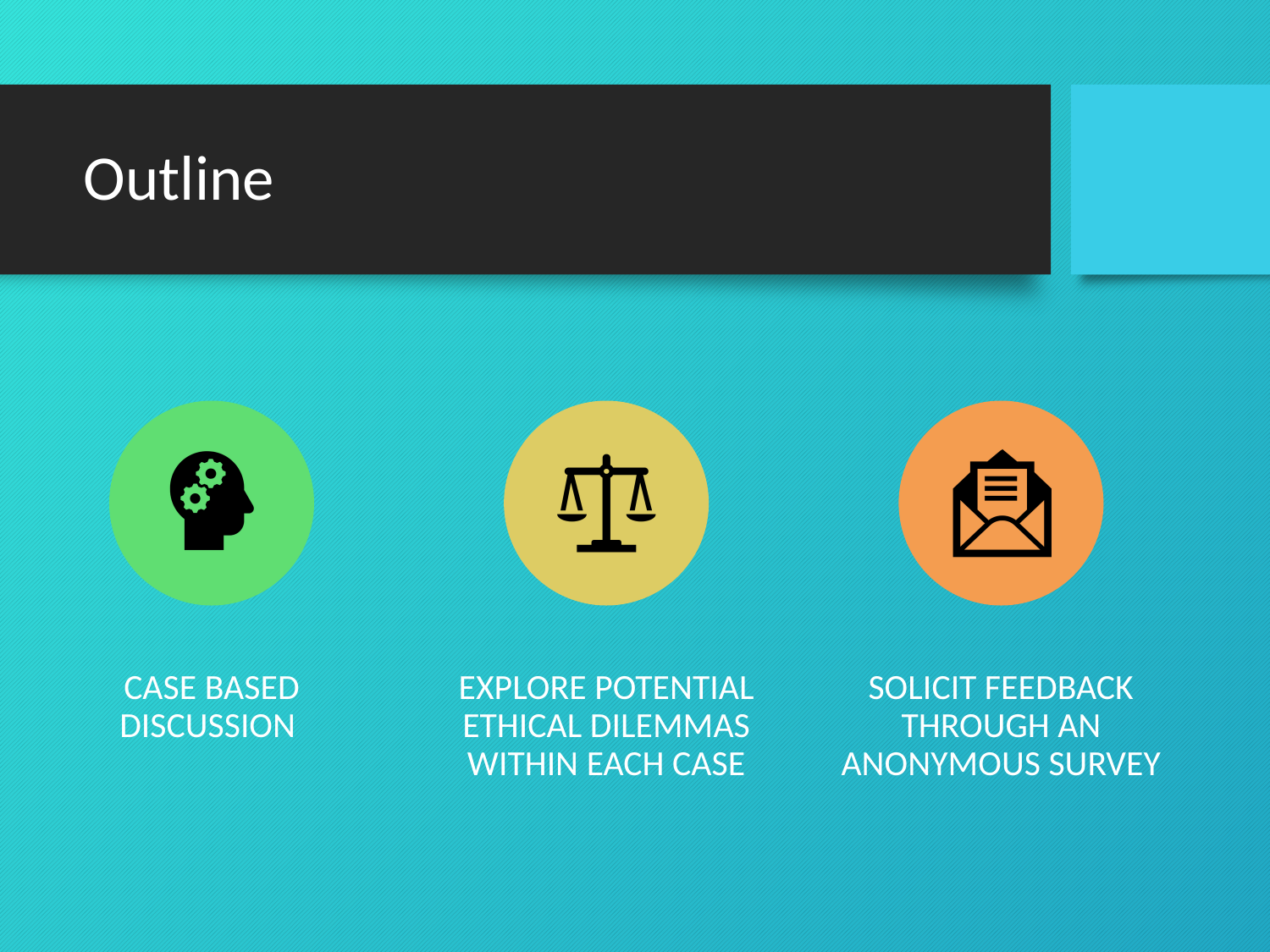

# Outline

## Slide 4
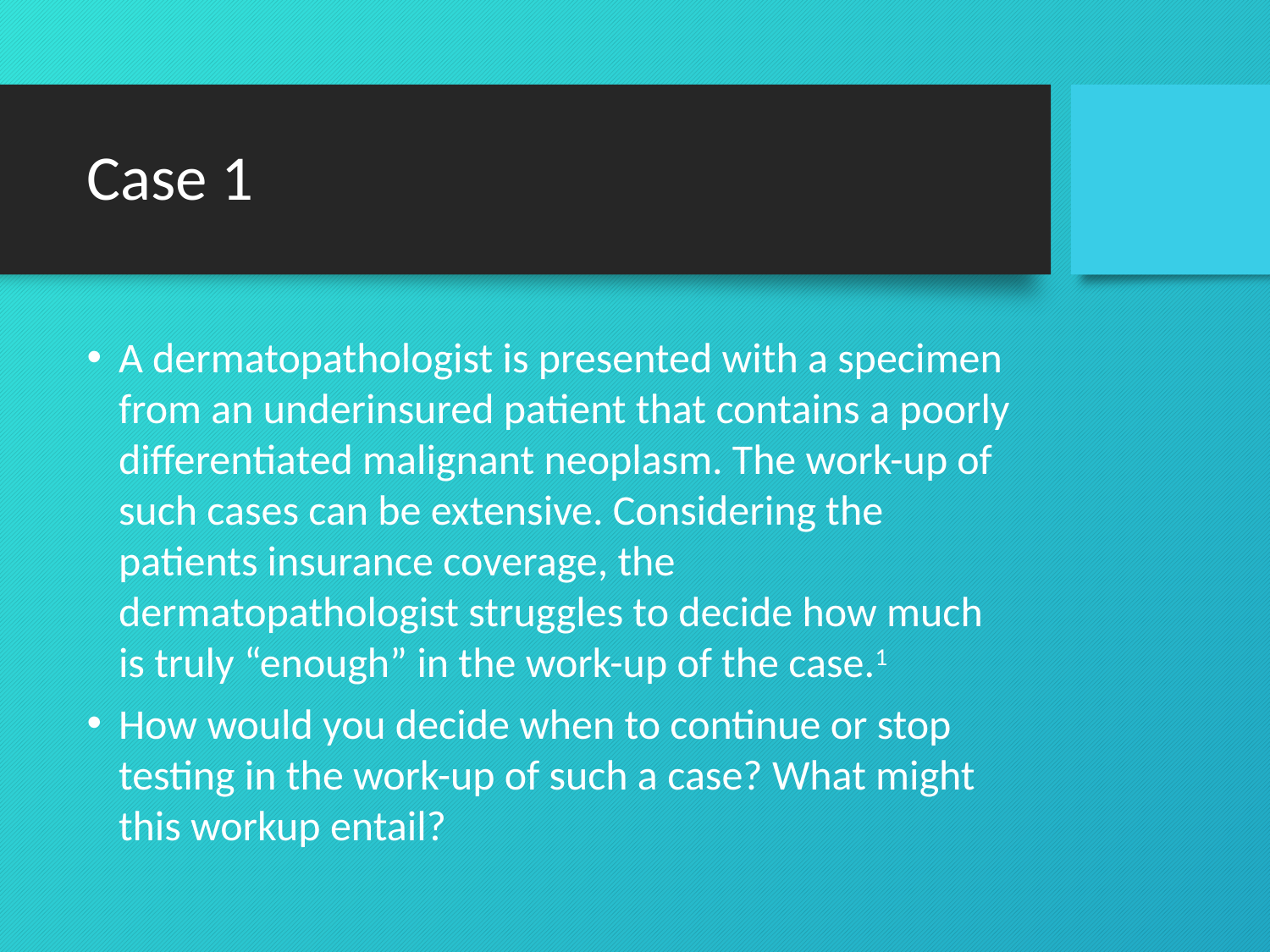

# Case 1
A dermatopathologist is presented with a specimen from an underinsured patient that contains a poorly differentiated malignant neoplasm. The work-up of such cases can be extensive. Considering the patients insurance coverage, the dermatopathologist struggles to decide how much is truly “enough” in the work-up of the case.1
How would you decide when to continue or stop testing in the work-up of such a case? What might this workup entail?

## Slide 5
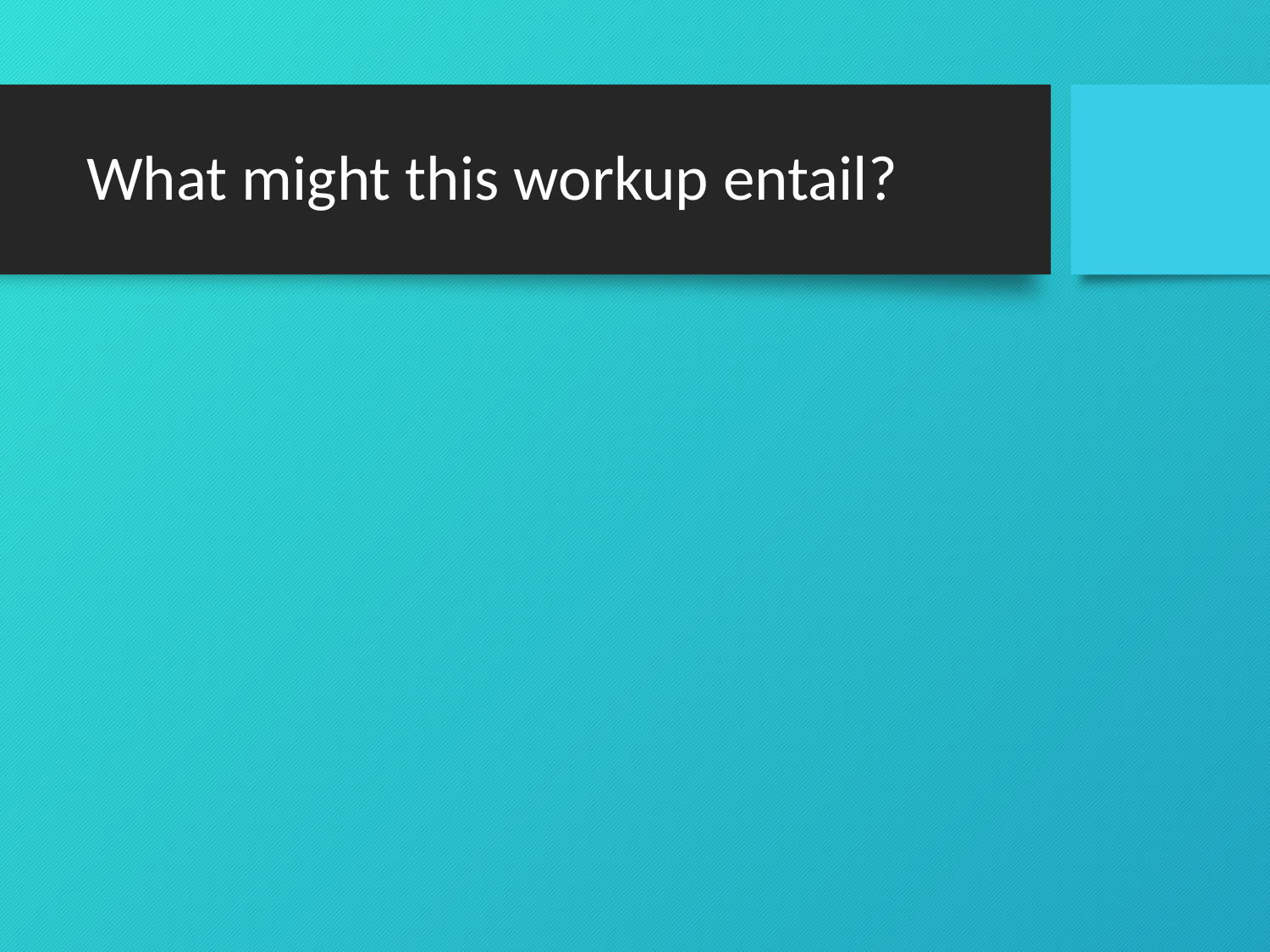

# What might this workup entail?

## Slide 6
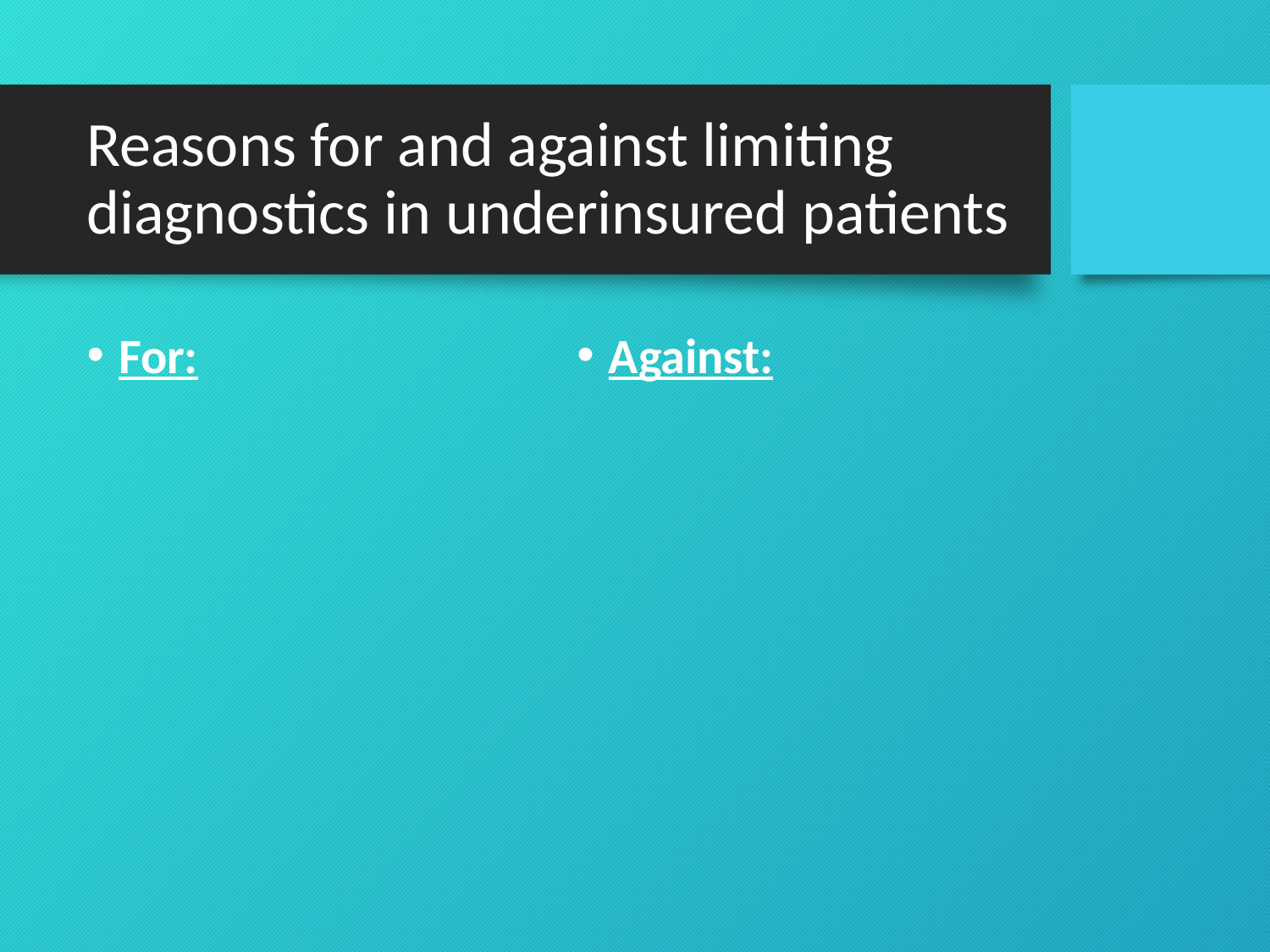

# Reasons for and against limiting diagnostics in underinsured patients
For:
Against:

## Slide 7
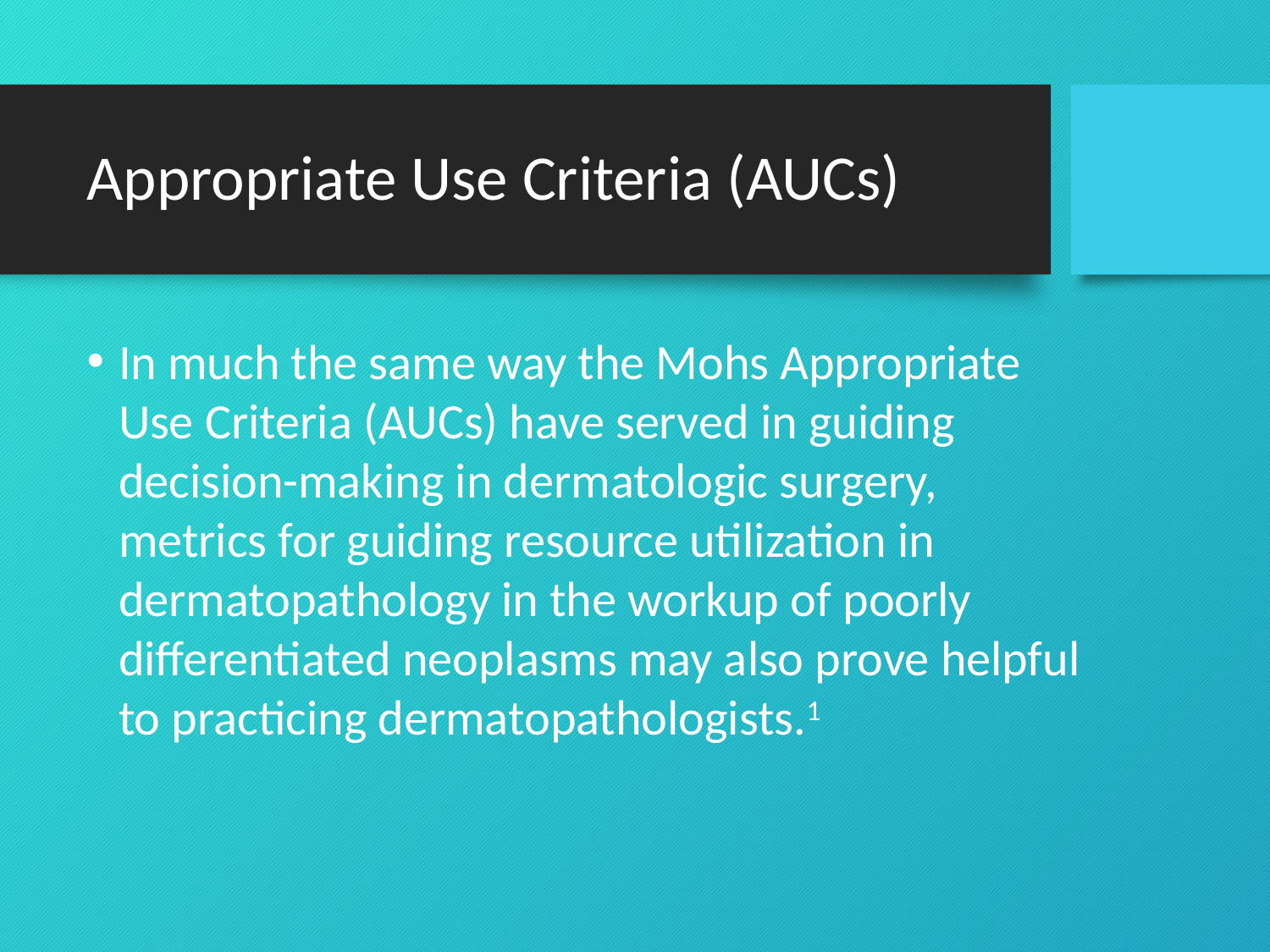

# Appropriate Use Criteria (AUCs)
In much the same way the Mohs Appropriate Use Criteria (AUCs) have served in guiding decision-making in dermatologic surgery, metrics for guiding resource utilization in dermatopathology in the workup of poorly differentiated neoplasms may also prove helpful to practicing dermatopathologists.1

## Slide 8
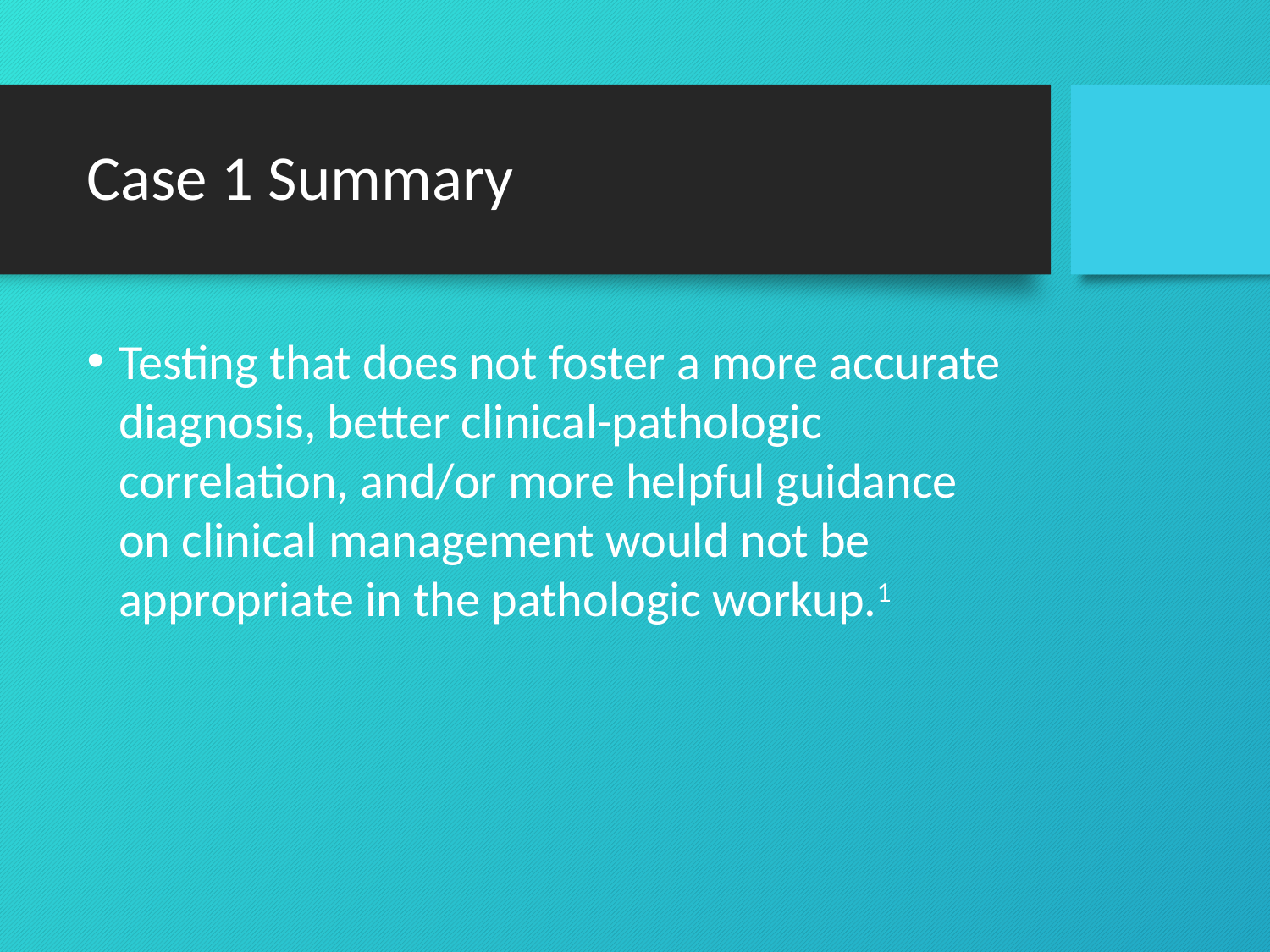

# Case 1 Summary
Testing that does not foster a more accurate diagnosis, better clinical-pathologic correlation, and/or more helpful guidance on clinical management would not be appropriate in the pathologic workup.1

## Slide 9
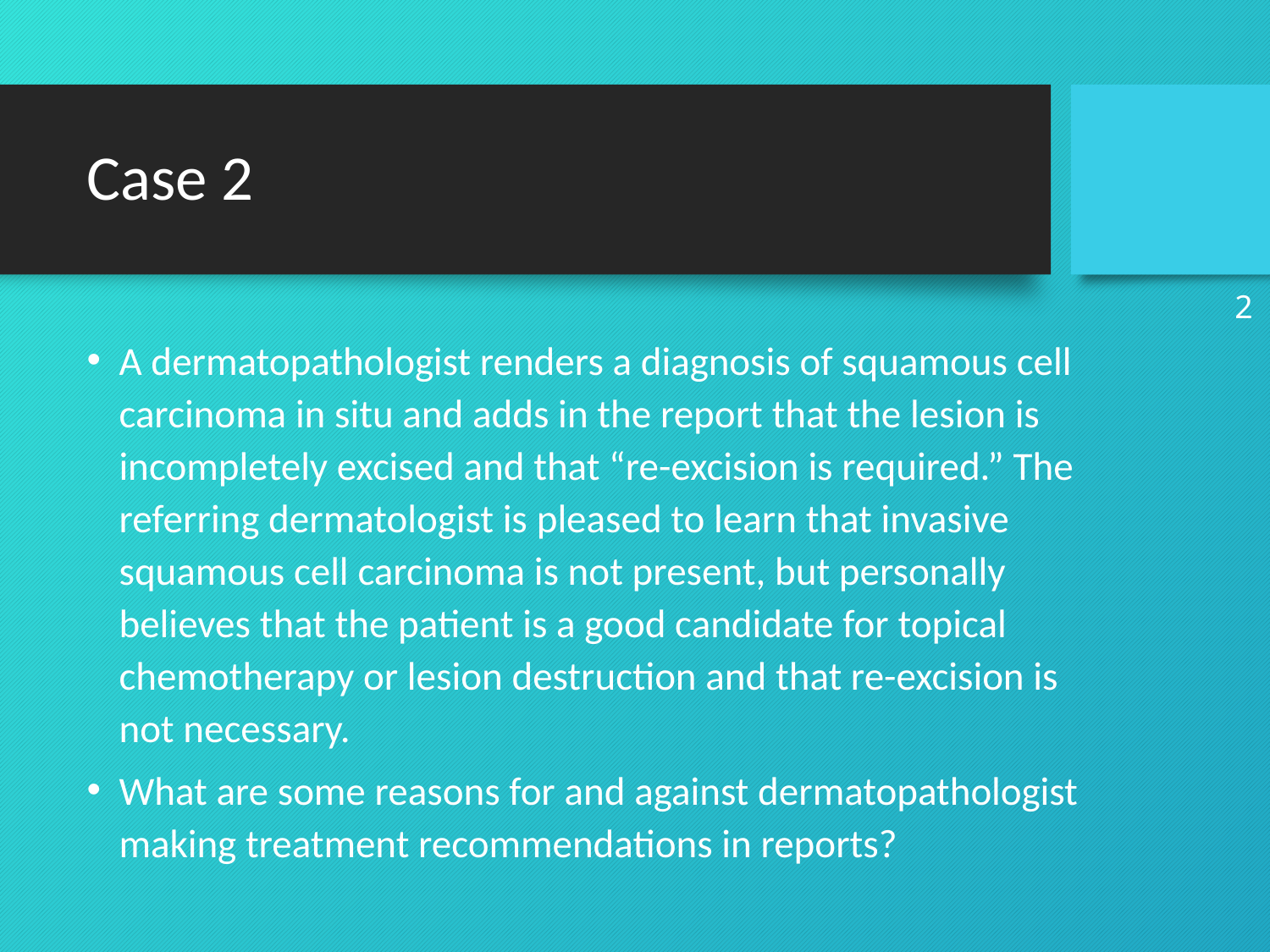

# Case 2
2
A dermatopathologist renders a diagnosis of squamous cell carcinoma in situ and adds in the report that the lesion is incompletely excised and that “re-excision is required.” The referring dermatologist is pleased to learn that invasive squamous cell carcinoma is not present, but personally believes that the patient is a good candidate for topical chemotherapy or lesion destruction and that re-excision is not necessary.
What are some reasons for and against dermatopathologist making treatment recommendations in reports?

## Slide 10
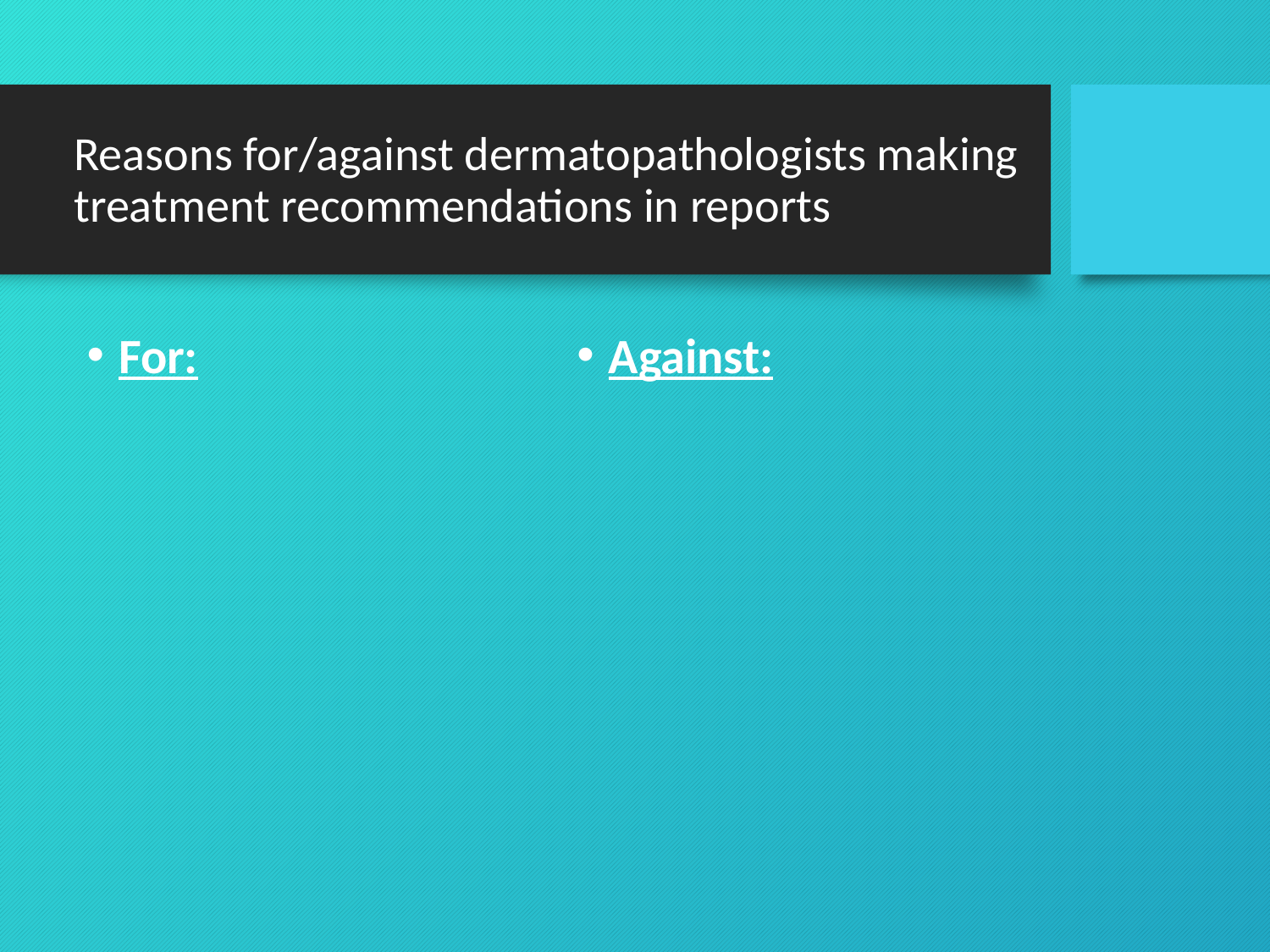

# Reasons for/against dermatopathologists making treatment recommendations in reports
For:
Against:

## Slide 11
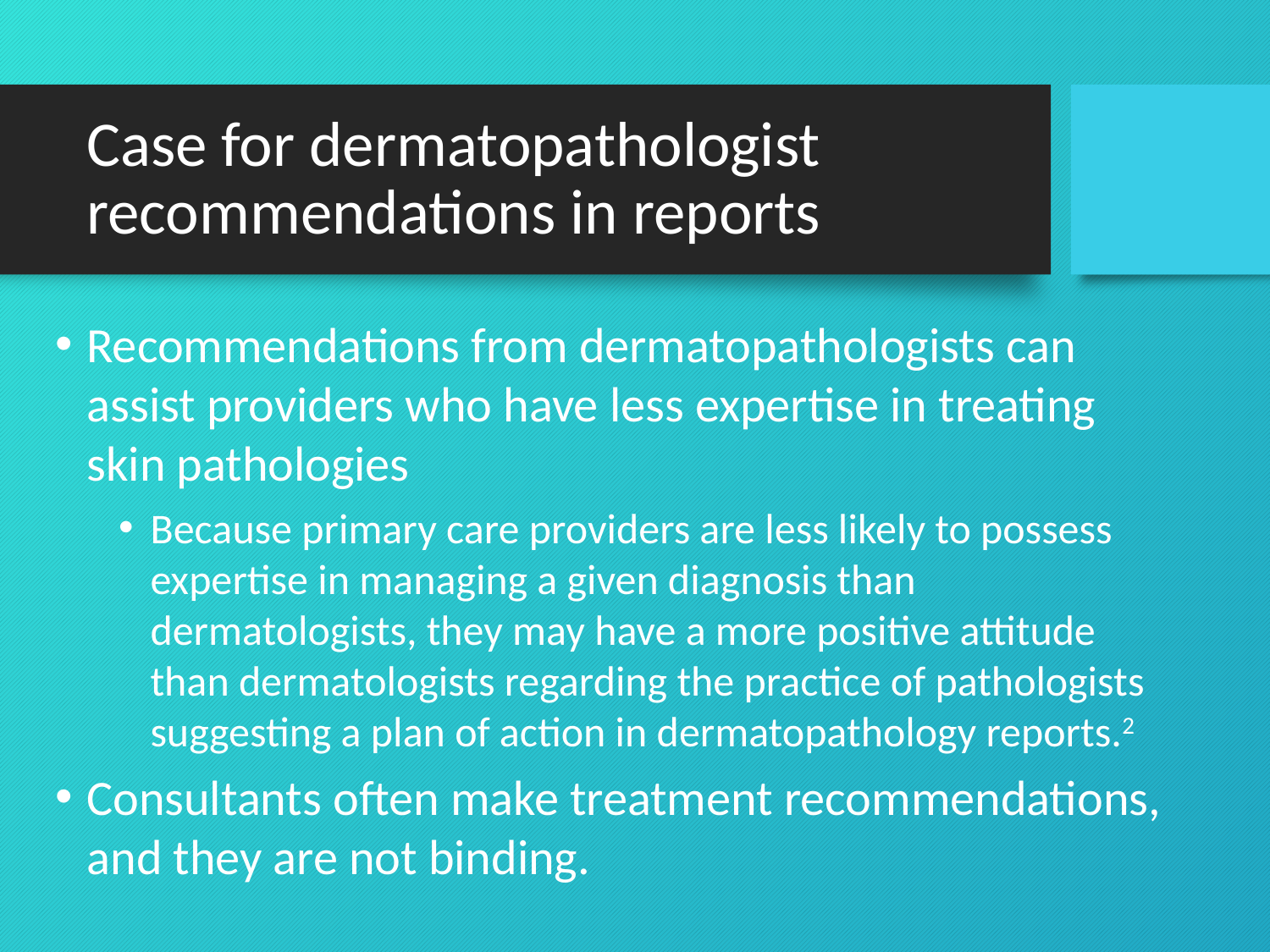

# Case for dermatopathologist recommendations in reports
Recommendations from dermatopathologists can assist providers who have less expertise in treating skin pathologies
Because primary care providers are less likely to possess expertise in managing a given diagnosis than dermatologists, they may have a more positive attitude than dermatologists regarding the practice of pathologists suggesting a plan of action in dermatopathology reports.2
Consultants often make treatment recommendations, and they are not binding.

## Slide 12
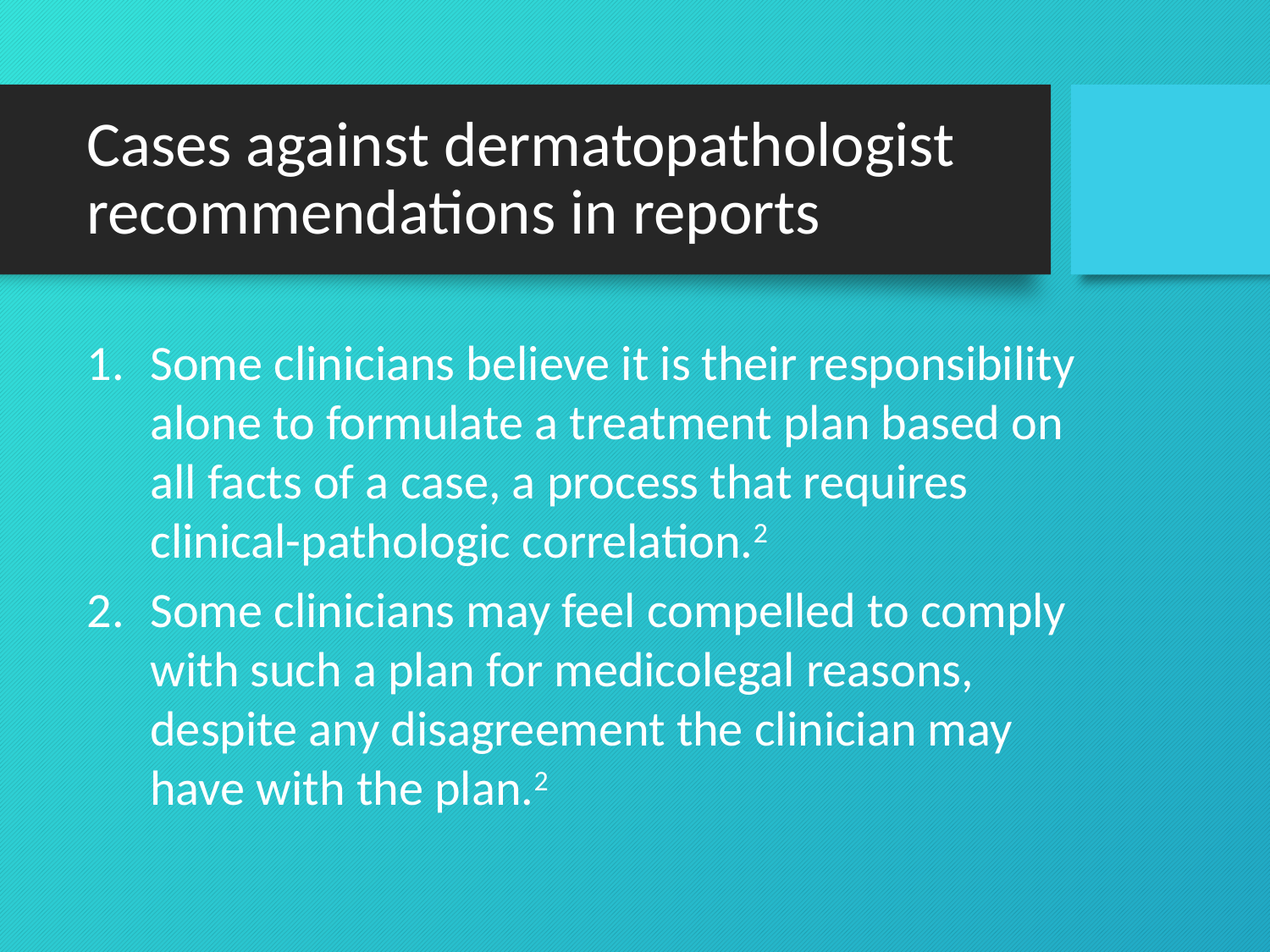

# Cases against dermatopathologist recommendations in reports
Some clinicians believe it is their responsibility alone to formulate a treatment plan based on all facts of a case, a process that requires clinical-pathologic correlation.2
Some clinicians may feel compelled to comply with such a plan for medicolegal reasons, despite any disagreement the clinician may have with the plan.2

## Slide 13
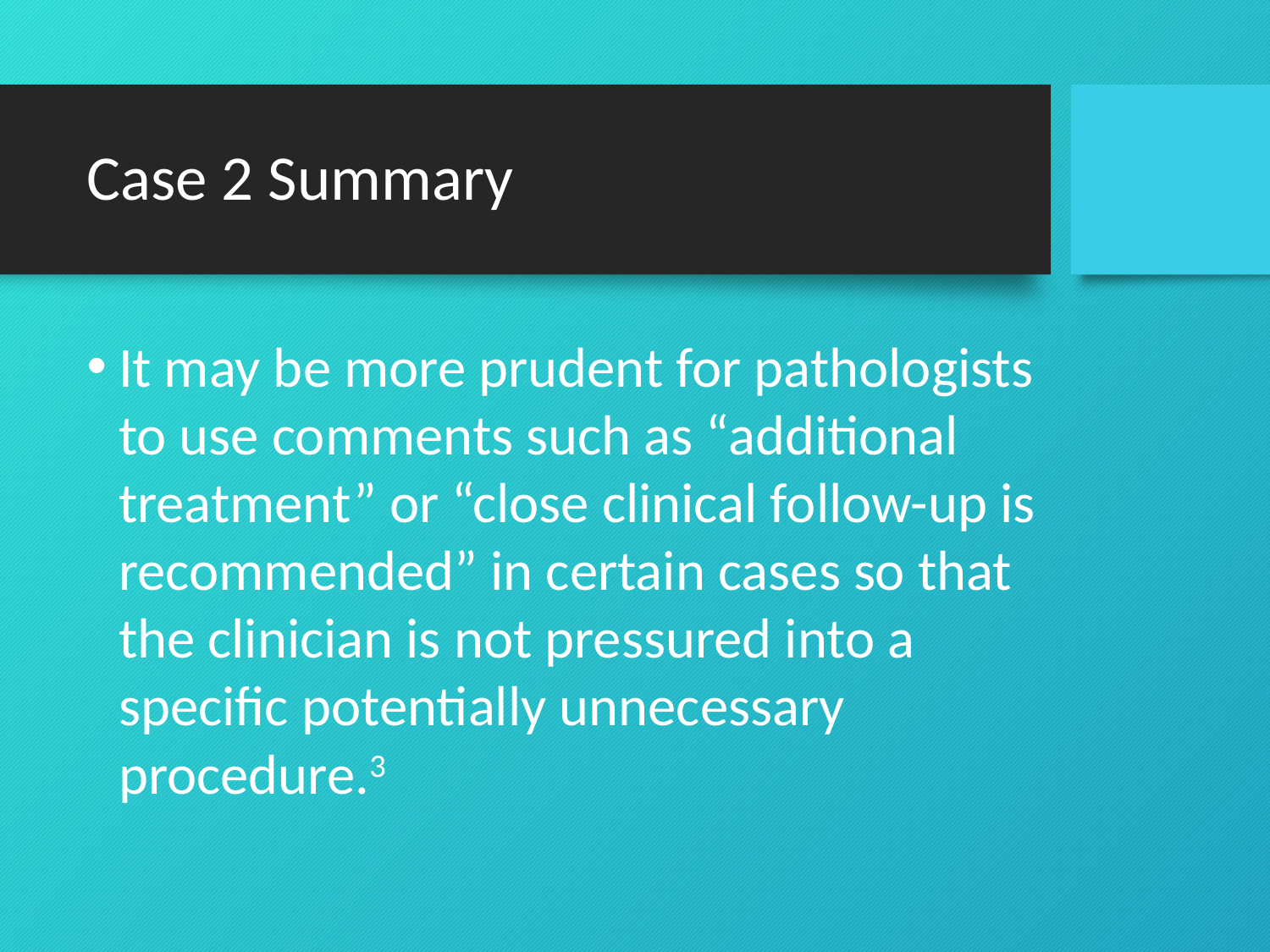

# Case 2 Summary
It may be more prudent for pathologists to use comments such as “additional treatment” or “close clinical follow-up is recommended” in certain cases so that the clinician is not pressured into a specific potentially unnecessary procedure.3

## Slide 14
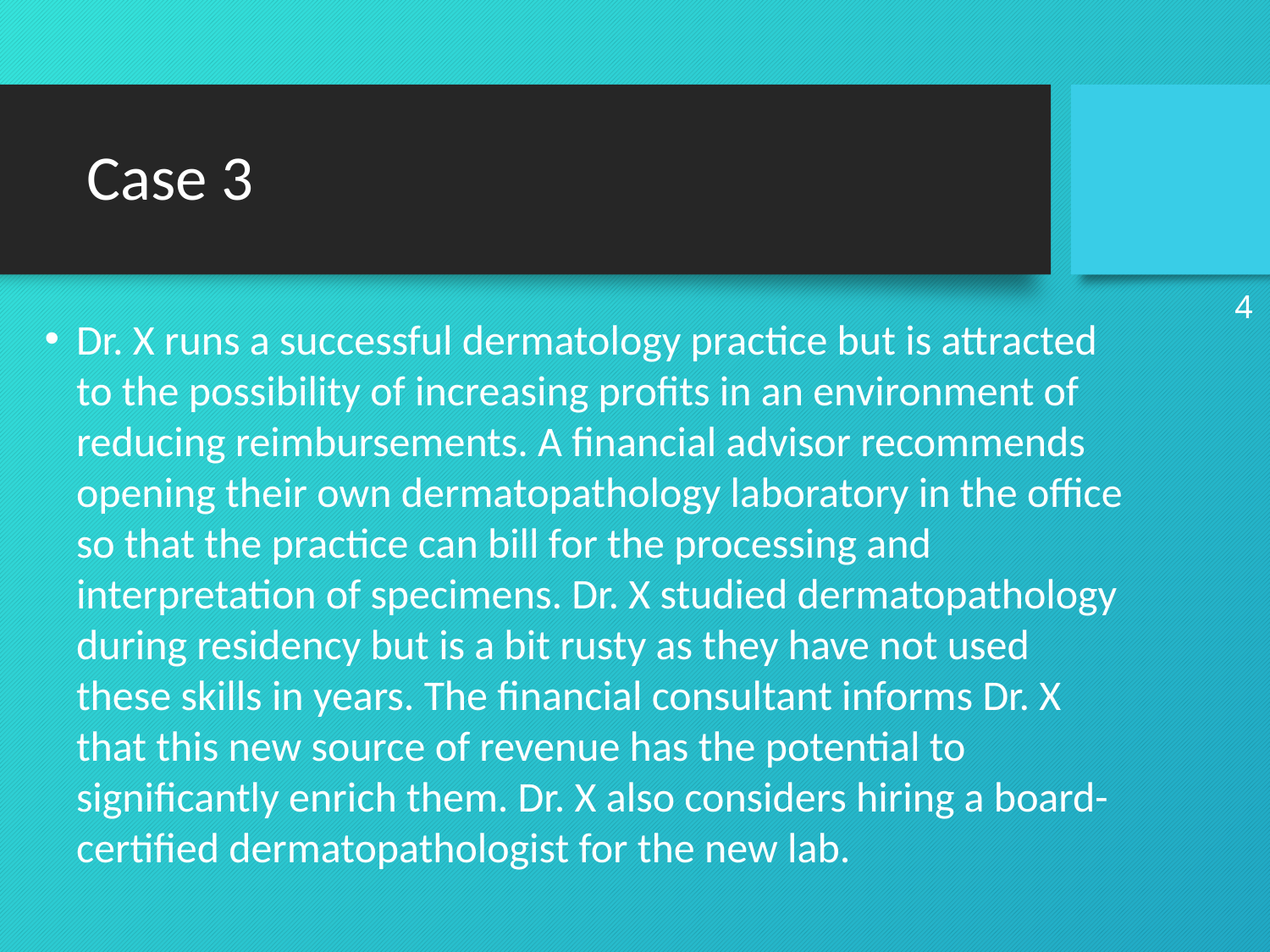

# Case 3
4
Dr. X runs a successful dermatology practice but is attracted to the possibility of increasing profits in an environment of reducing reimbursements. A financial advisor recommends opening their own dermatopathology laboratory in the office so that the practice can bill for the processing and interpretation of specimens. Dr. X studied dermatopathology during residency but is a bit rusty as they have not used these skills in years. The financial consultant informs Dr. X that this new source of revenue has the potential to significantly enrich them. Dr. X also considers hiring a board-certified dermatopathologist for the new lab.

## Slide 15
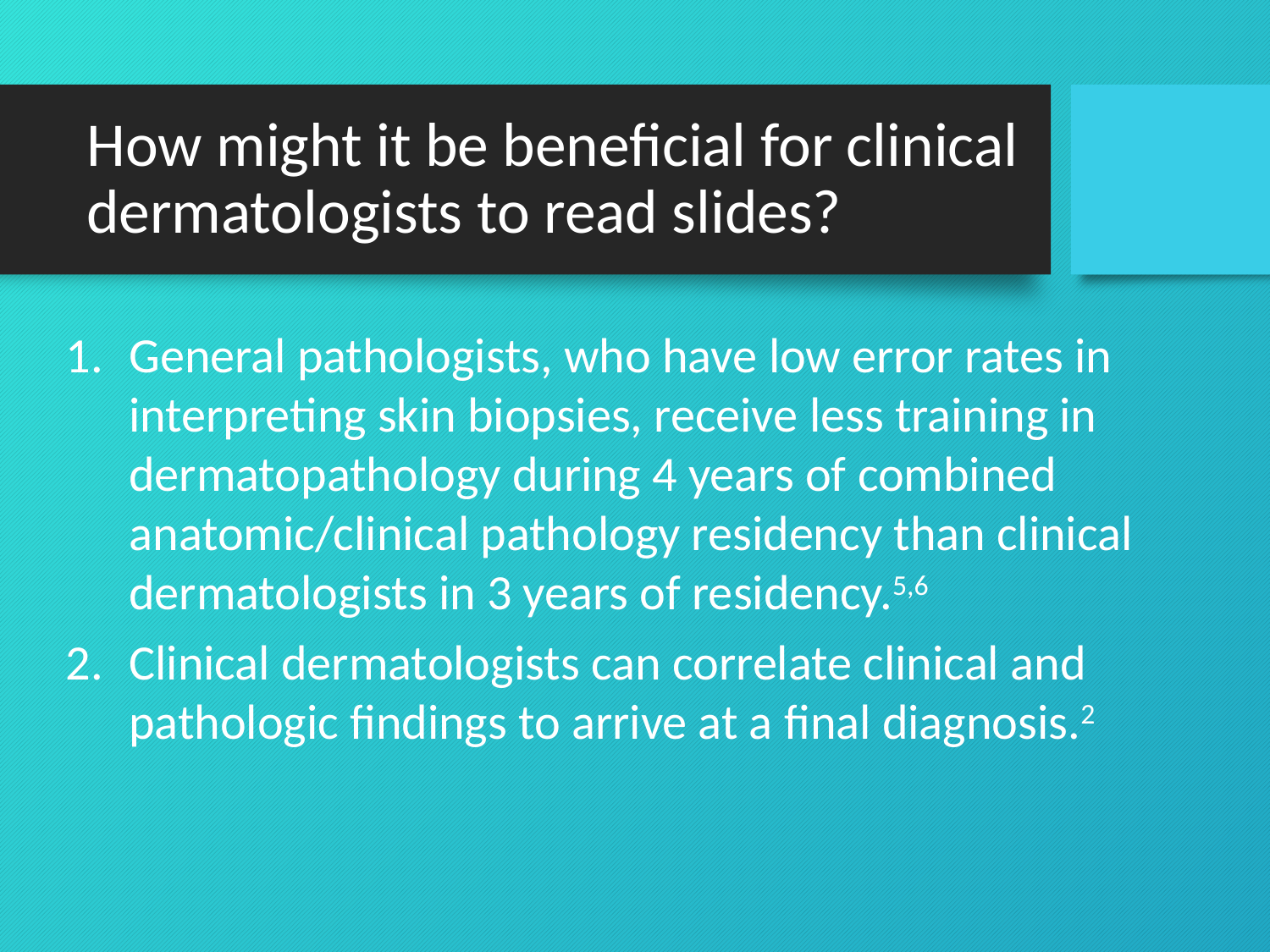

# How might it be beneficial for clinical dermatologists to read slides?
General pathologists, who have low error rates in interpreting skin biopsies, receive less training in dermatopathology during 4 years of combined anatomic/clinical pathology residency than clinical dermatologists in 3 years of residency.5,6
Clinical dermatologists can correlate clinical and pathologic findings to arrive at a final diagnosis.2

## Slide 16
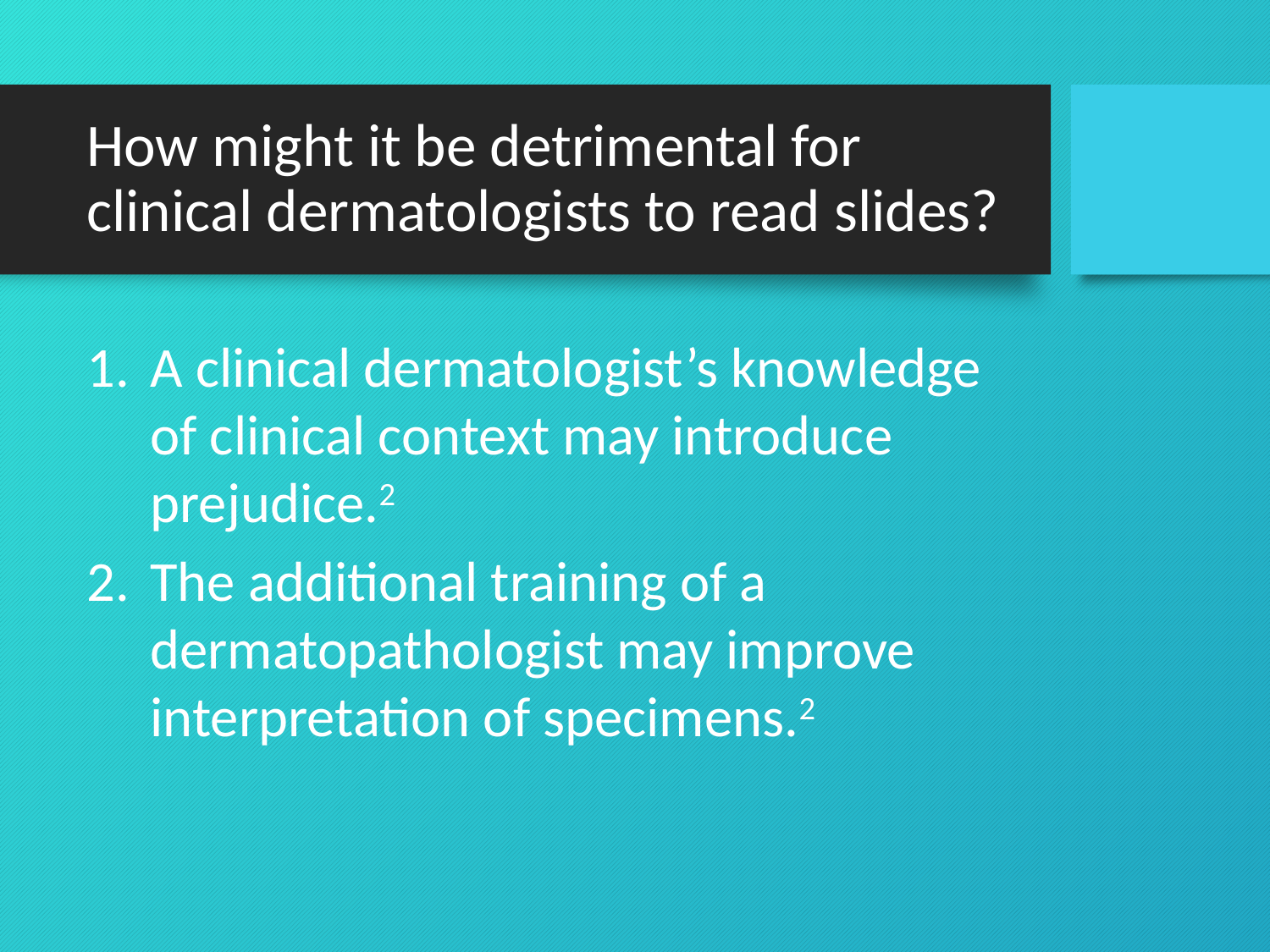

# How might it be detrimental for clinical dermatologists to read slides?
A clinical dermatologist’s knowledge of clinical context may introduce prejudice.2
The additional training of a dermatopathologist may improve interpretation of specimens.2

## Slide 17
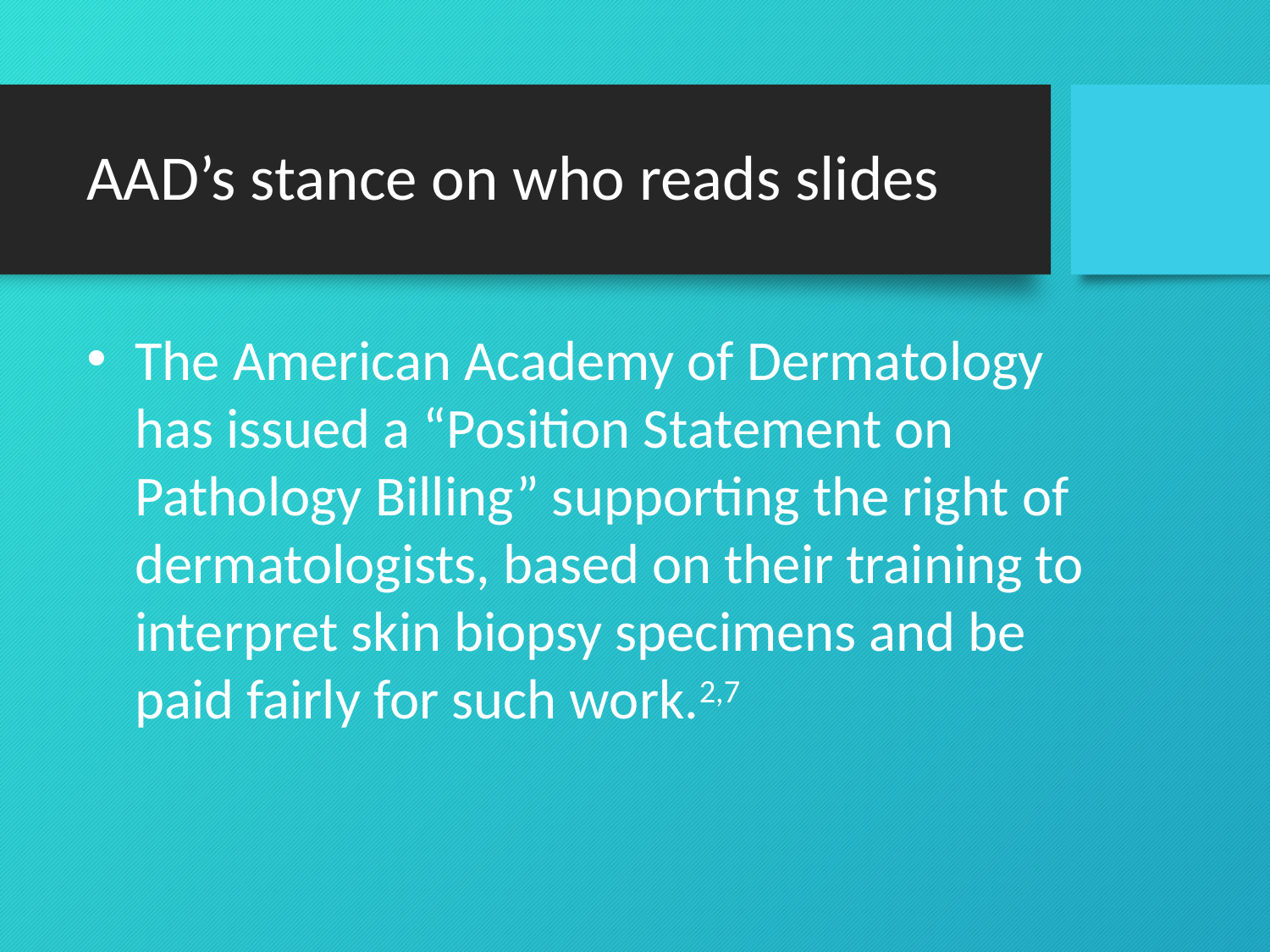

# AAD’s stance on who reads slides
The American Academy of Dermatology has issued a “Position Statement on Pathology Billing” supporting the right of dermatologists, based on their training to interpret skin biopsy specimens and be paid fairly for such work.2,7

## Slide 18
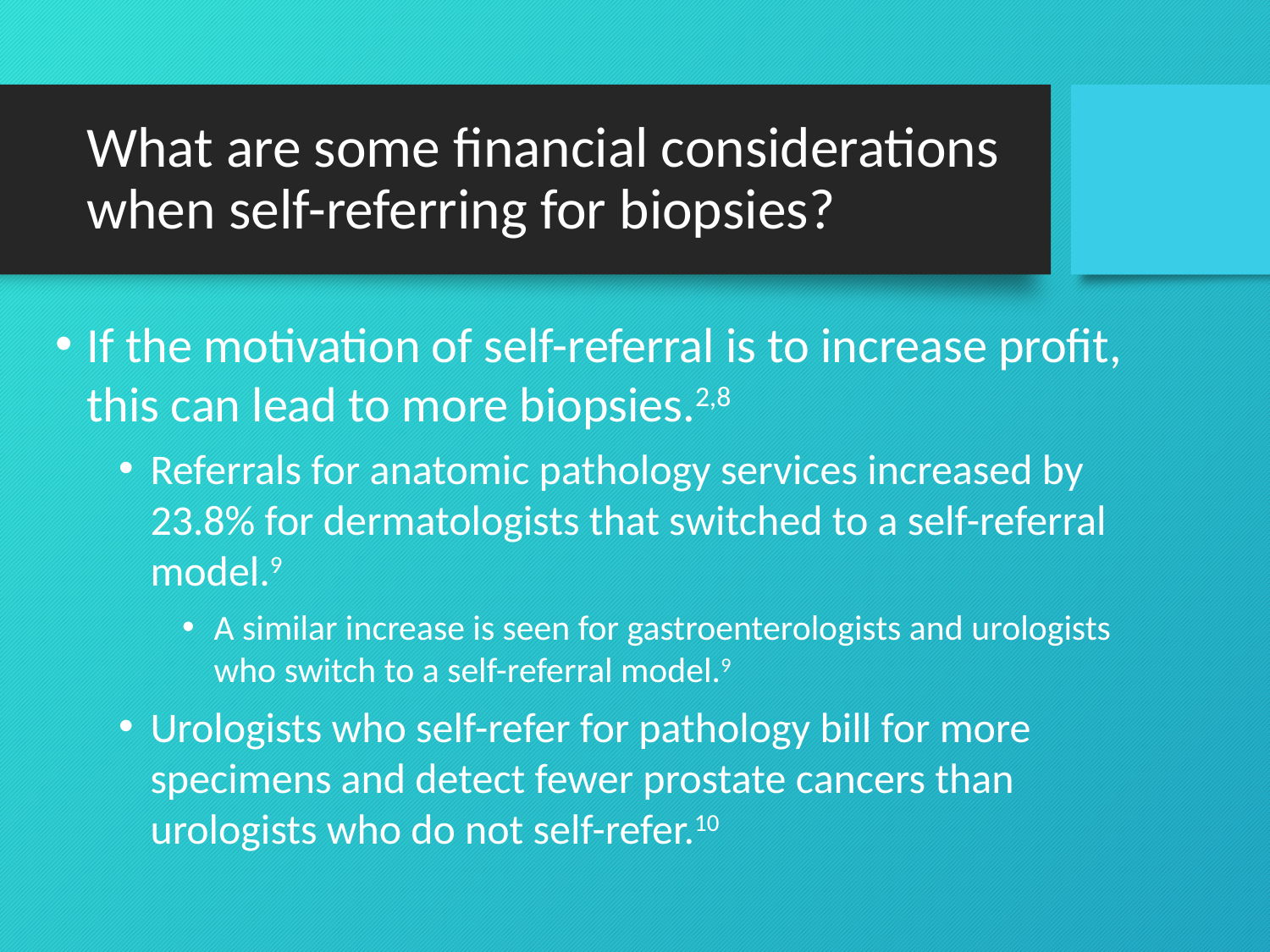

# What are some financial considerations when self-referring for biopsies?
If the motivation of self-referral is to increase profit, this can lead to more biopsies.2,8
Referrals for anatomic pathology services increased by 23.8% for dermatologists that switched to a self-referral model.9
A similar increase is seen for gastroenterologists and urologists who switch to a self-referral model.9
Urologists who self-refer for pathology bill for more specimens and detect fewer prostate cancers than urologists who do not self-refer.10

## Slide 19
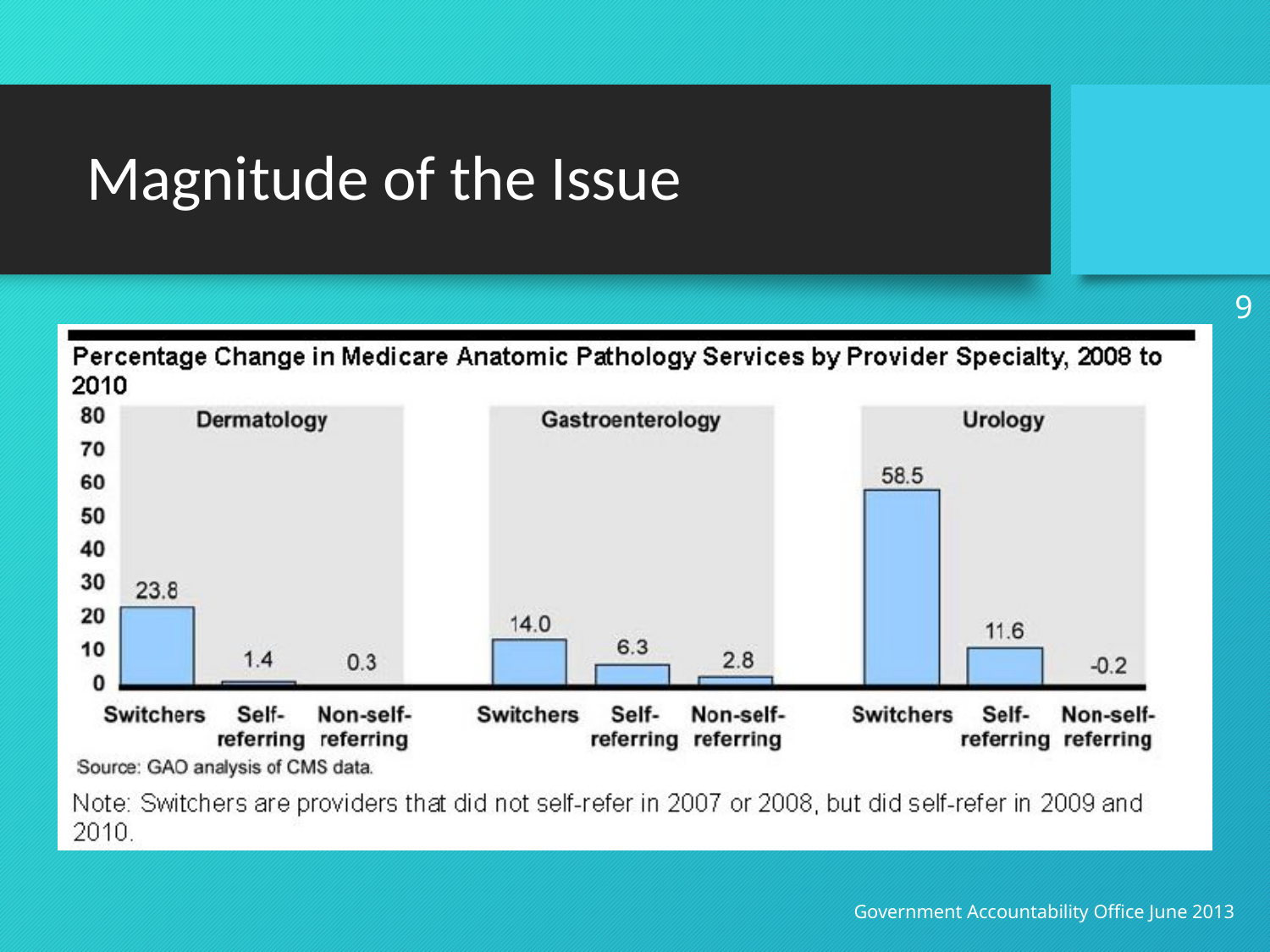

# Magnitude of the Issue
9
Government Accountability Office June 2013

## Slide 20
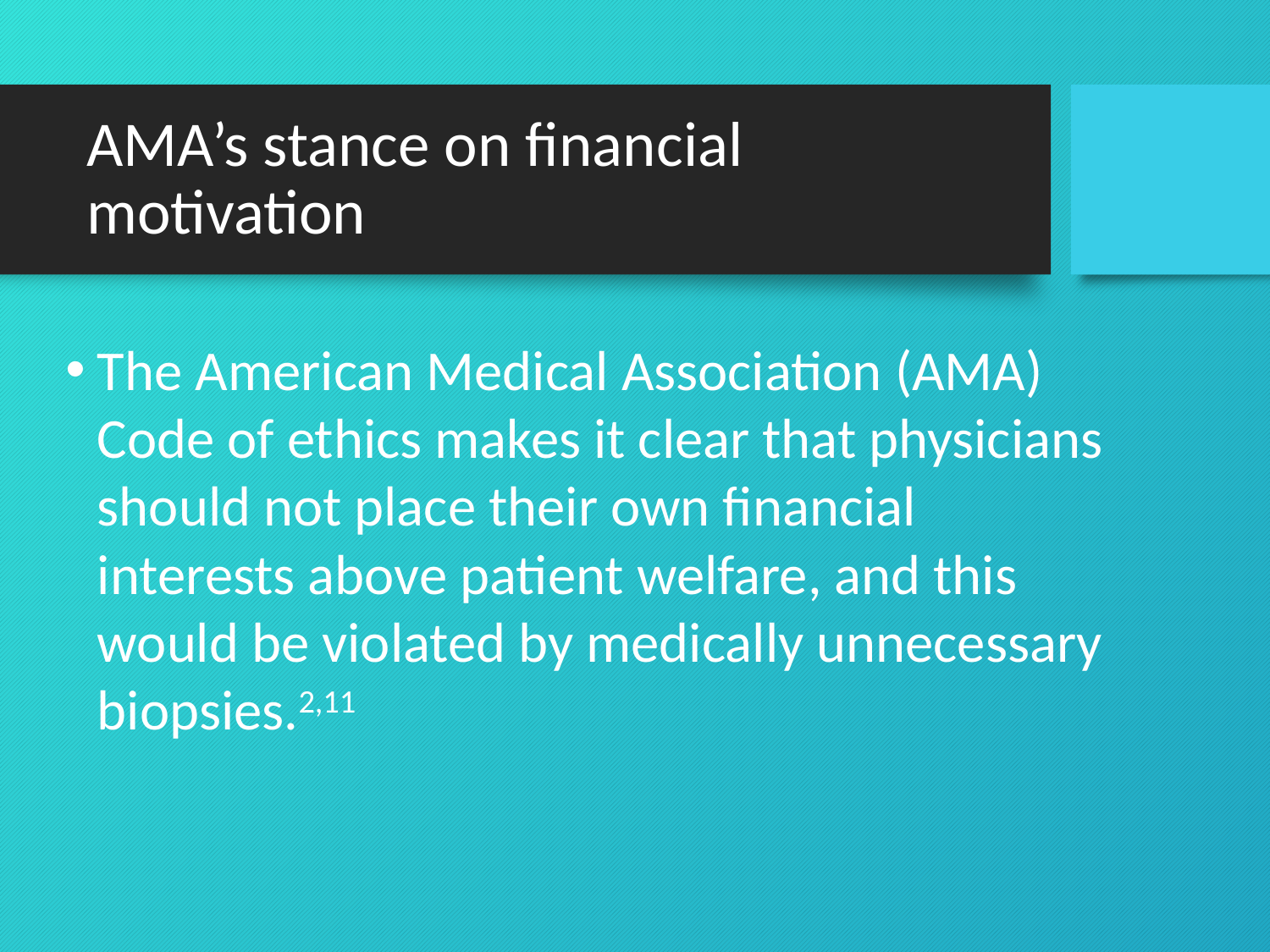

# AMA’s stance on financial motivation
The American Medical Association (AMA) Code of ethics makes it clear that physicians should not place their own financial interests above patient welfare, and this would be violated by medically unnecessary biopsies.2,11

## Slide 21
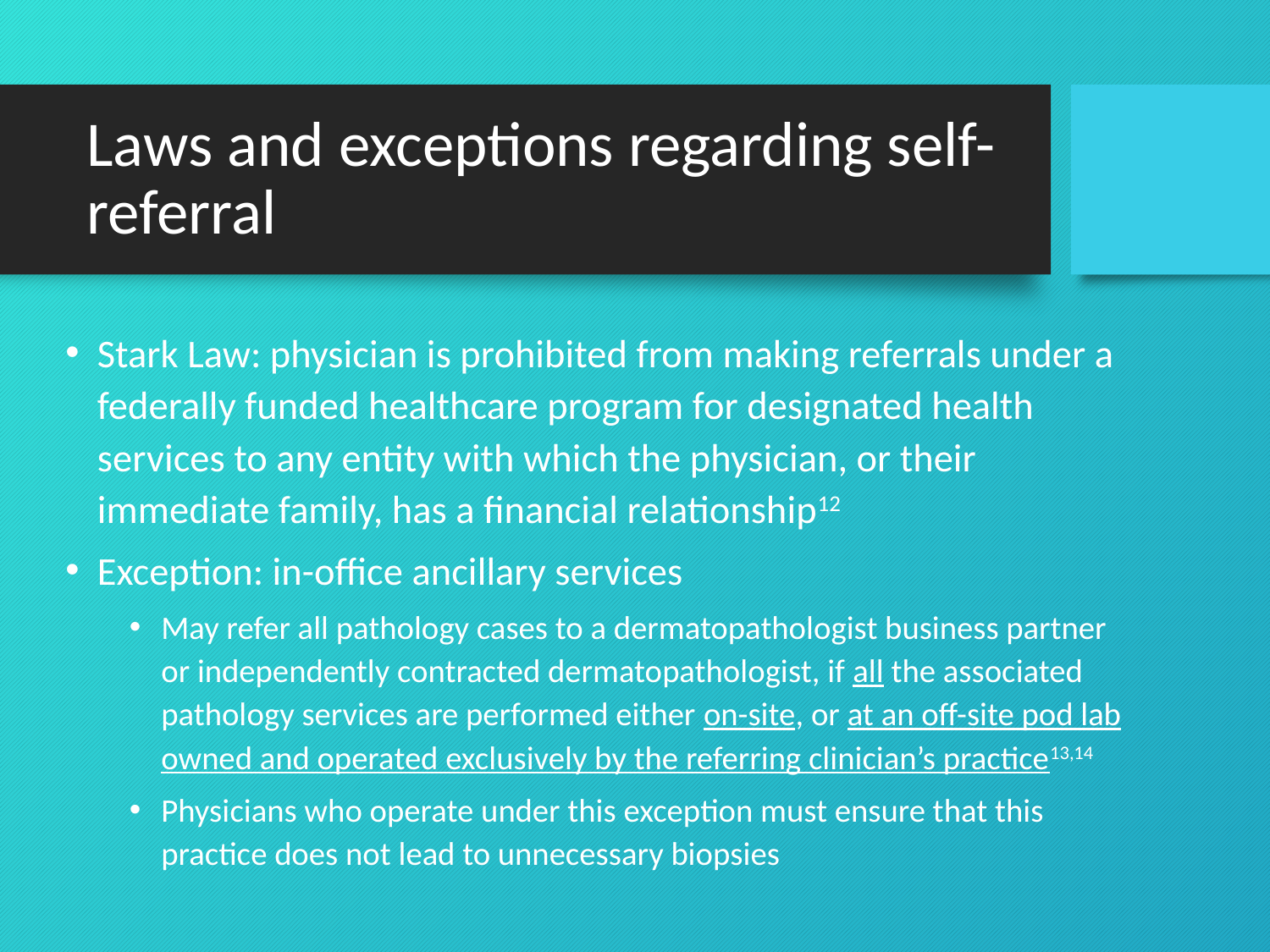

# Laws and exceptions regarding self-referral
Stark Law: physician is prohibited from making referrals under a federally funded healthcare program for designated health services to any entity with which the physician, or their immediate family, has a financial relationship12
Exception: in-office ancillary services
May refer all pathology cases to a dermatopathologist business partner or independently contracted dermatopathologist, if all the associated pathology services are performed either on-site, or at an off-site pod lab owned and operated exclusively by the referring clinician’s practice13,14
Physicians who operate under this exception must ensure that this practice does not lead to unnecessary biopsies

## Slide 22
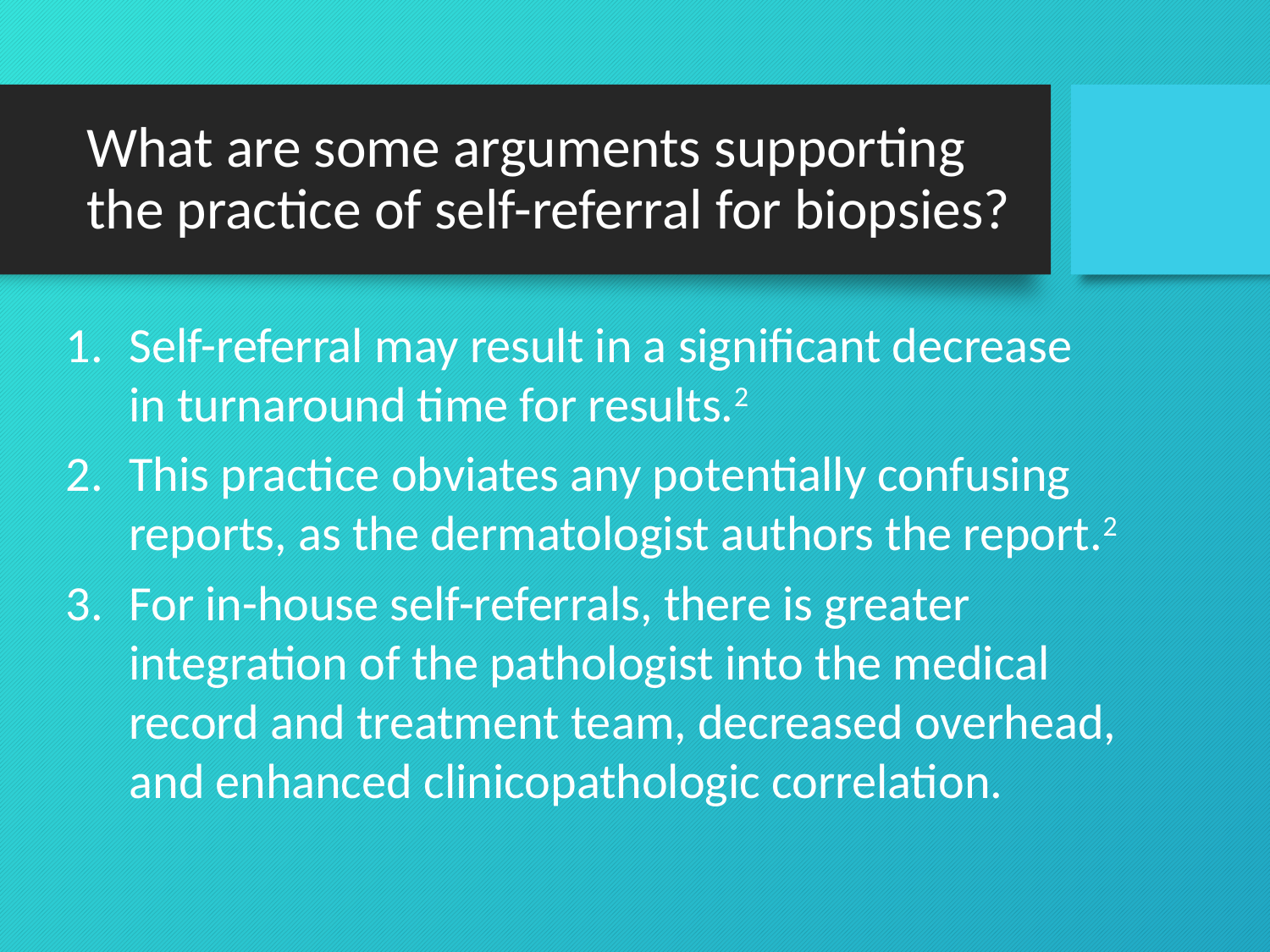

# What are some arguments supporting the practice of self-referral for biopsies?
Self-referral may result in a significant decrease in turnaround time for results.2
This practice obviates any potentially confusing reports, as the dermatologist authors the report.2
For in-house self-referrals, there is greater integration of the pathologist into the medical record and treatment team, decreased overhead, and enhanced clinicopathologic correlation.

## Slide 23
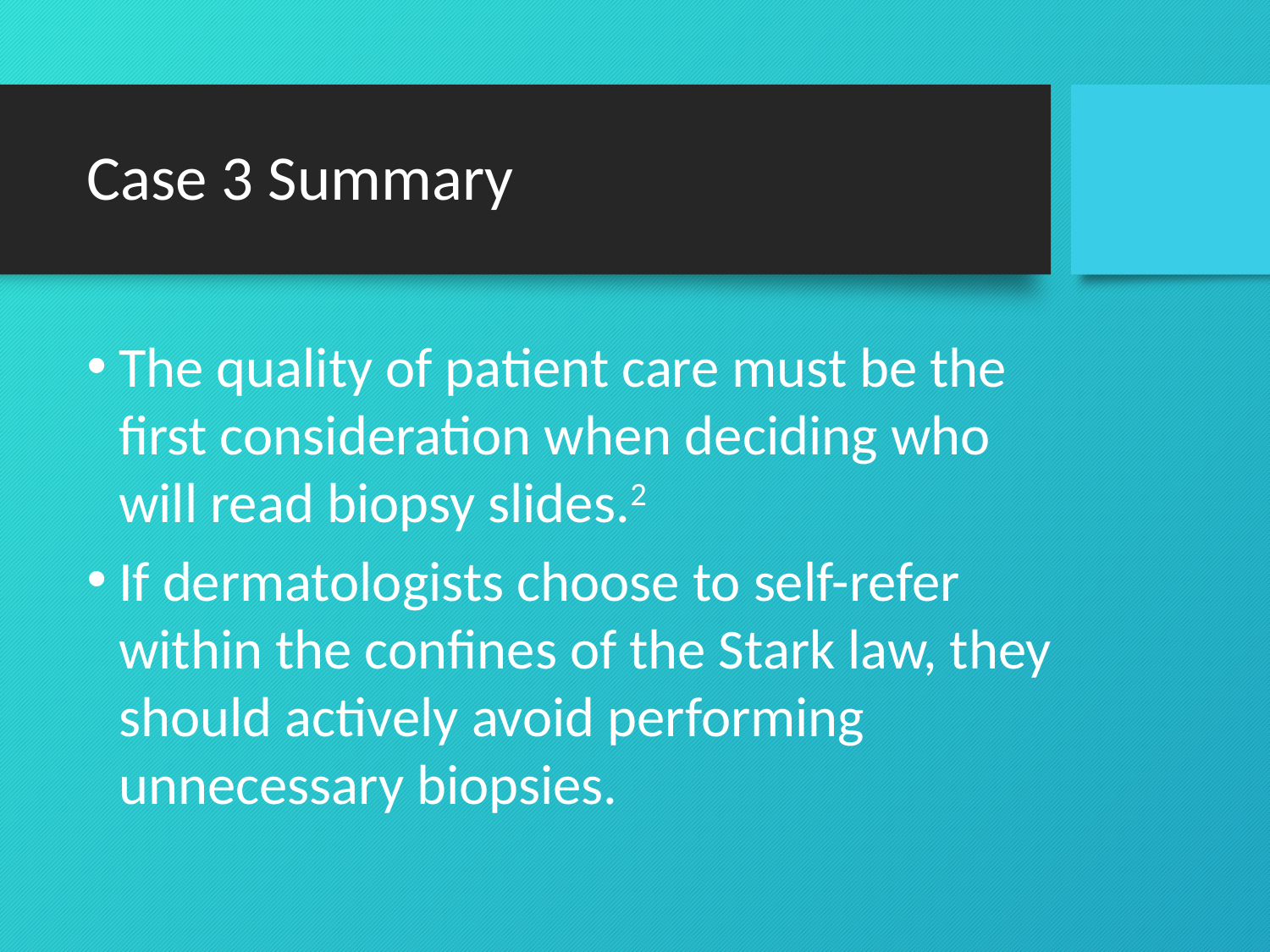

# Case 3 Summary
The quality of patient care must be the first consideration when deciding who will read biopsy slides.2
If dermatologists choose to self-refer within the confines of the Stark law, they should actively avoid performing unnecessary biopsies.

## Slide 24
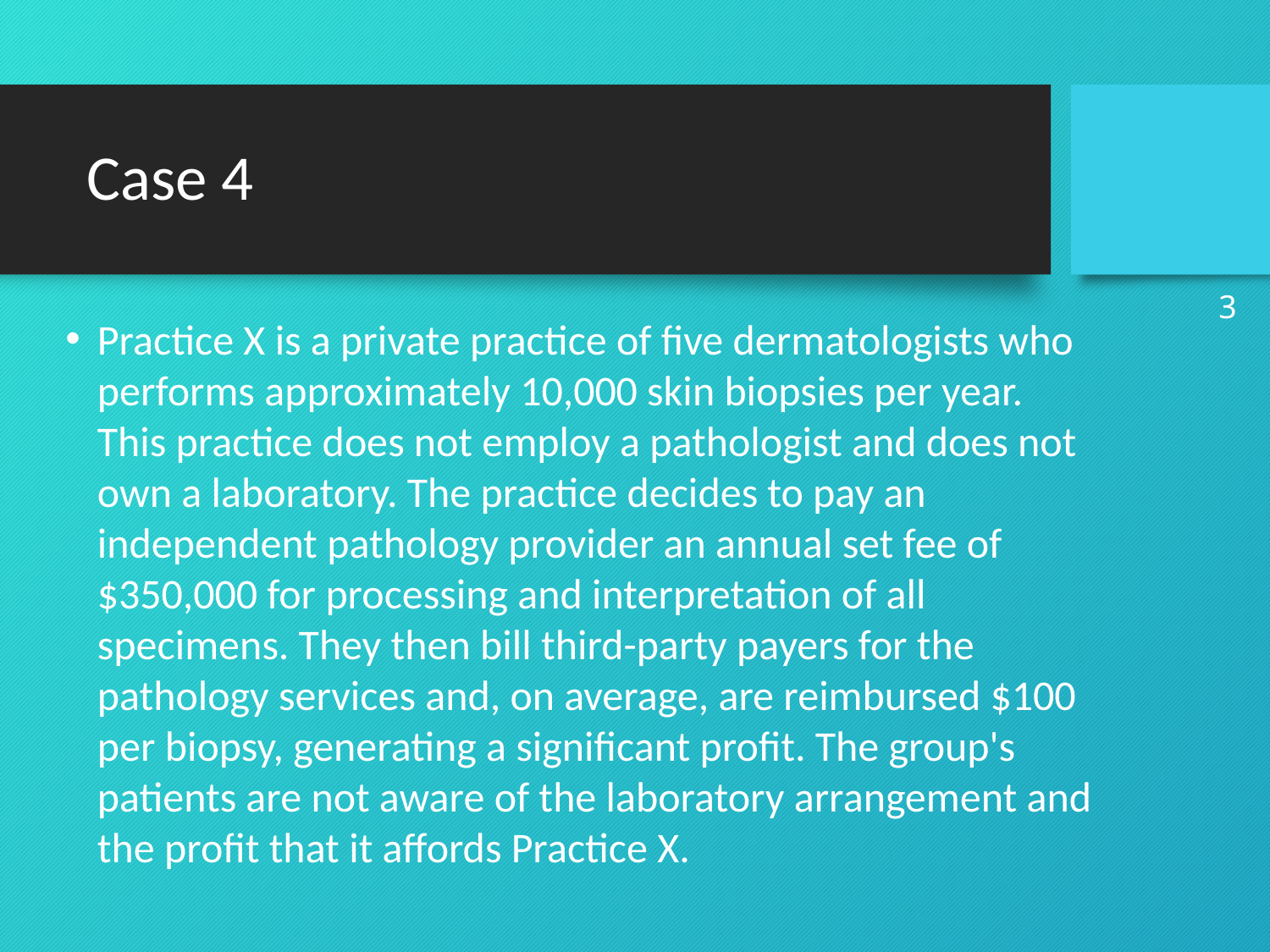

# Case 4
3
Practice X is a private practice of five dermatologists who performs approximately 10,000 skin biopsies per year. This practice does not employ a pathologist and does not own a laboratory. The practice decides to pay an independent pathology provider an annual set fee of $350,000 for processing and interpretation of all specimens. They then bill third-party payers for the pathology services and, on average, are reimbursed $100 per biopsy, generating a significant profit. The group's patients are not aware of the laboratory arrangement and the profit that it affords Practice X.

## Slide 25
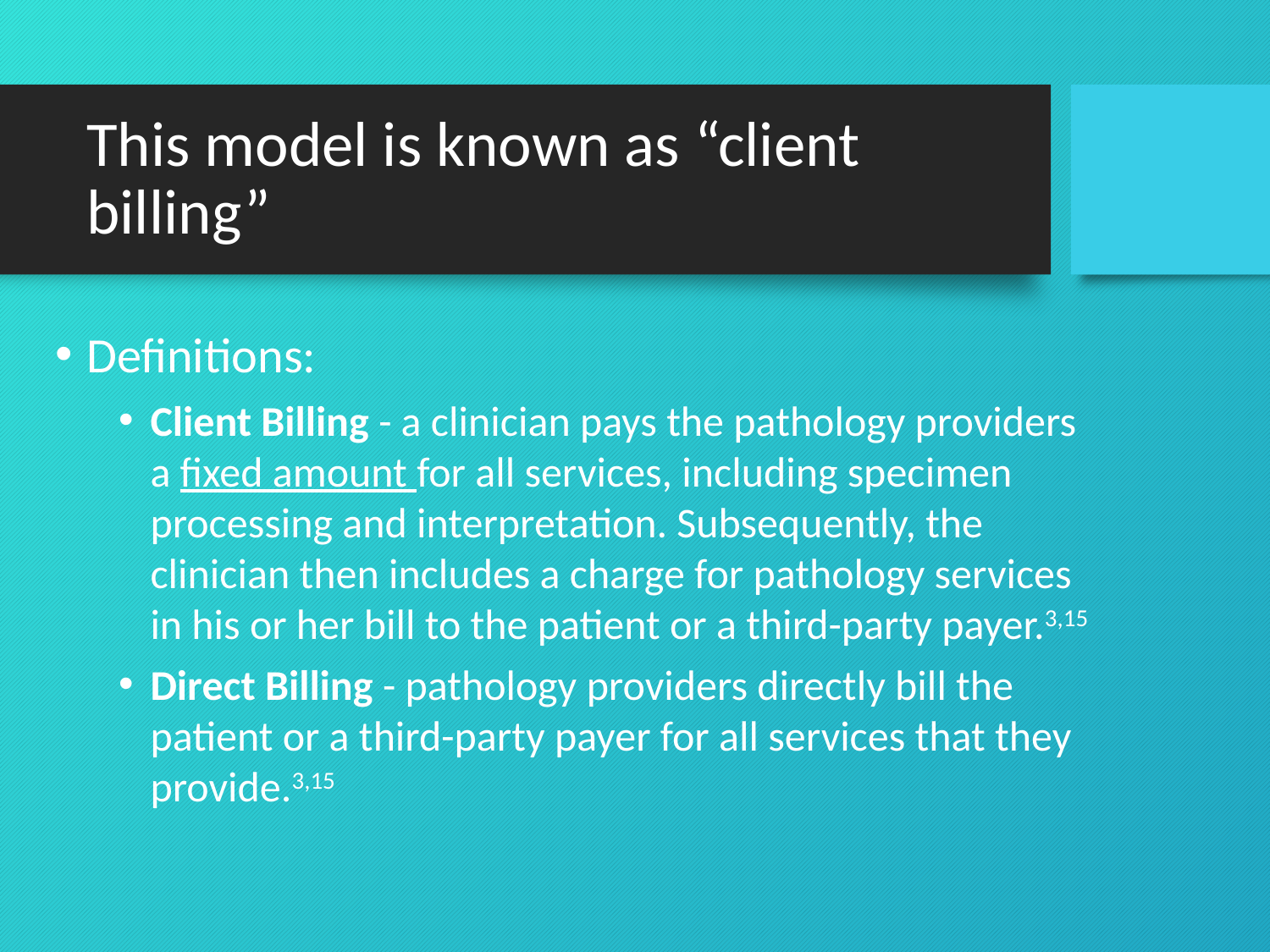

# This model is known as “client billing”
Definitions:
Client Billing - a clinician pays the pathology providers a fixed amount for all services, including specimen processing and interpretation. Subsequently, the clinician then includes a charge for pathology services in his or her bill to the patient or a third-party payer.3,15
Direct Billing - pathology providers directly bill the patient or a third-party payer for all services that they provide.3,15

## Slide 26
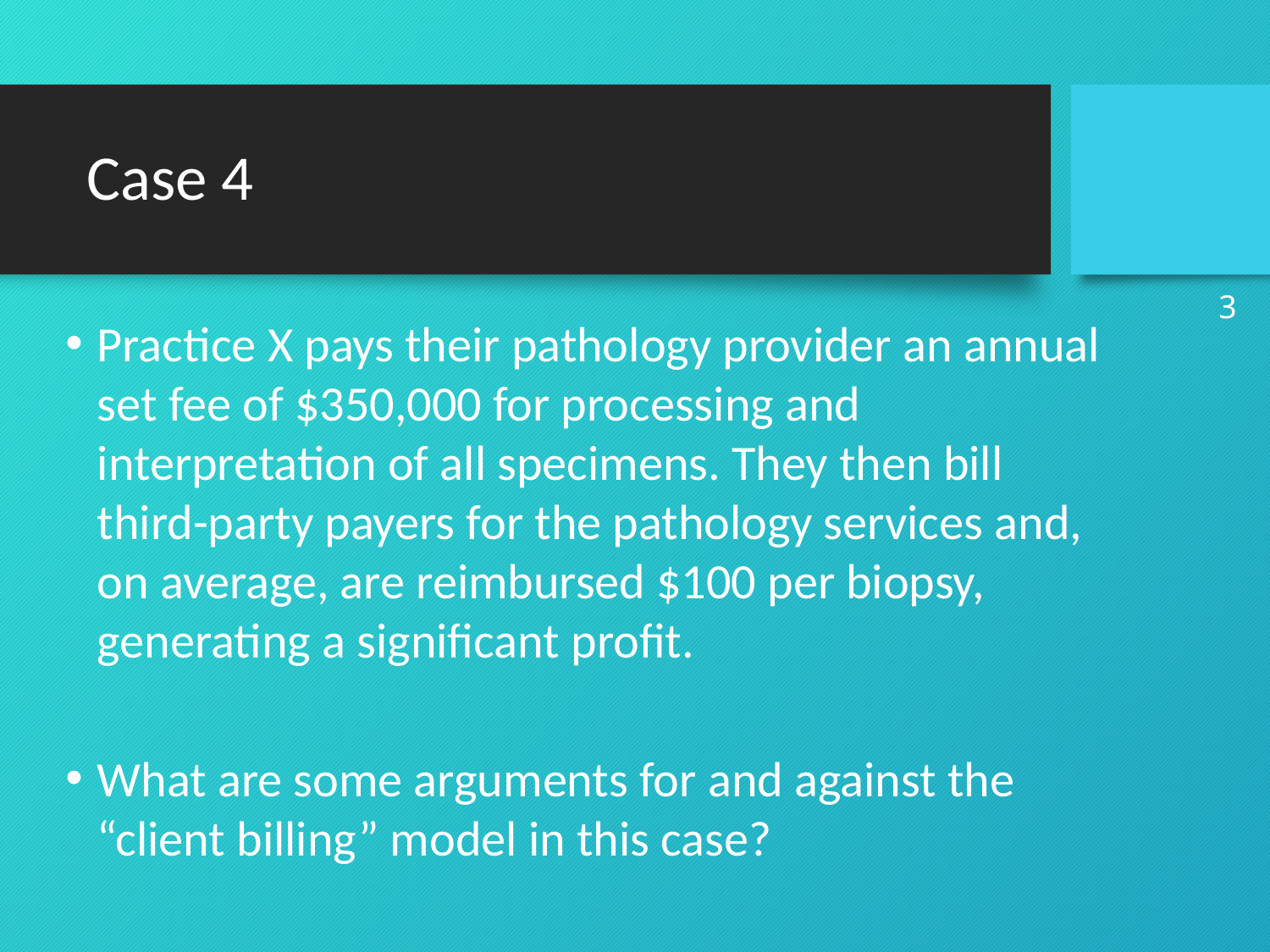

# Case 4
3
Practice X pays their pathology provider an annual set fee of $350,000 for processing and interpretation of all specimens. They then bill third-party payers for the pathology services and, on average, are reimbursed $100 per biopsy, generating a significant profit.
What are some arguments for and against the “client billing” model in this case?

## Slide 27
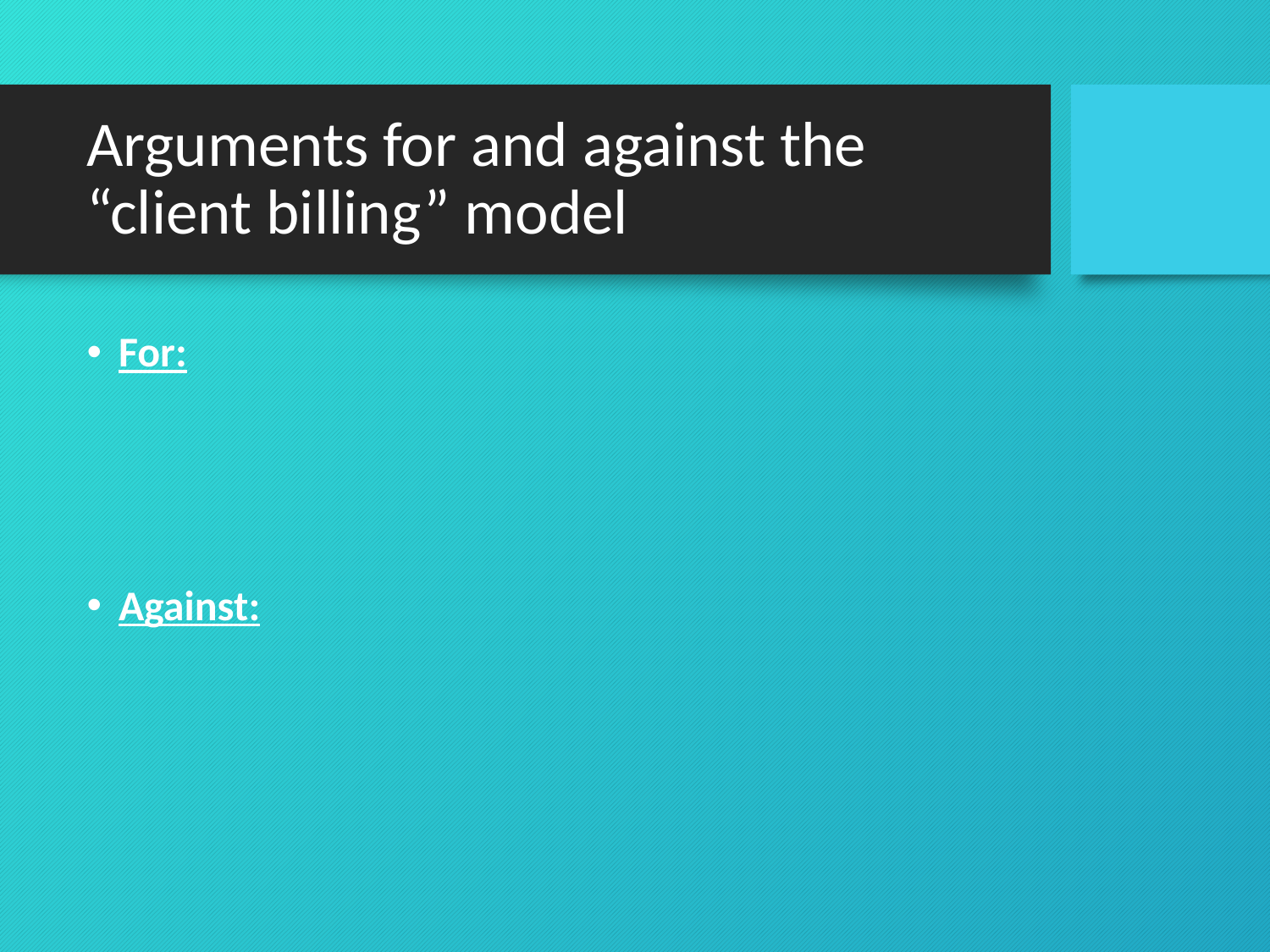

# Arguments for and against the “client billing” model
For:
Against:

## Slide 28
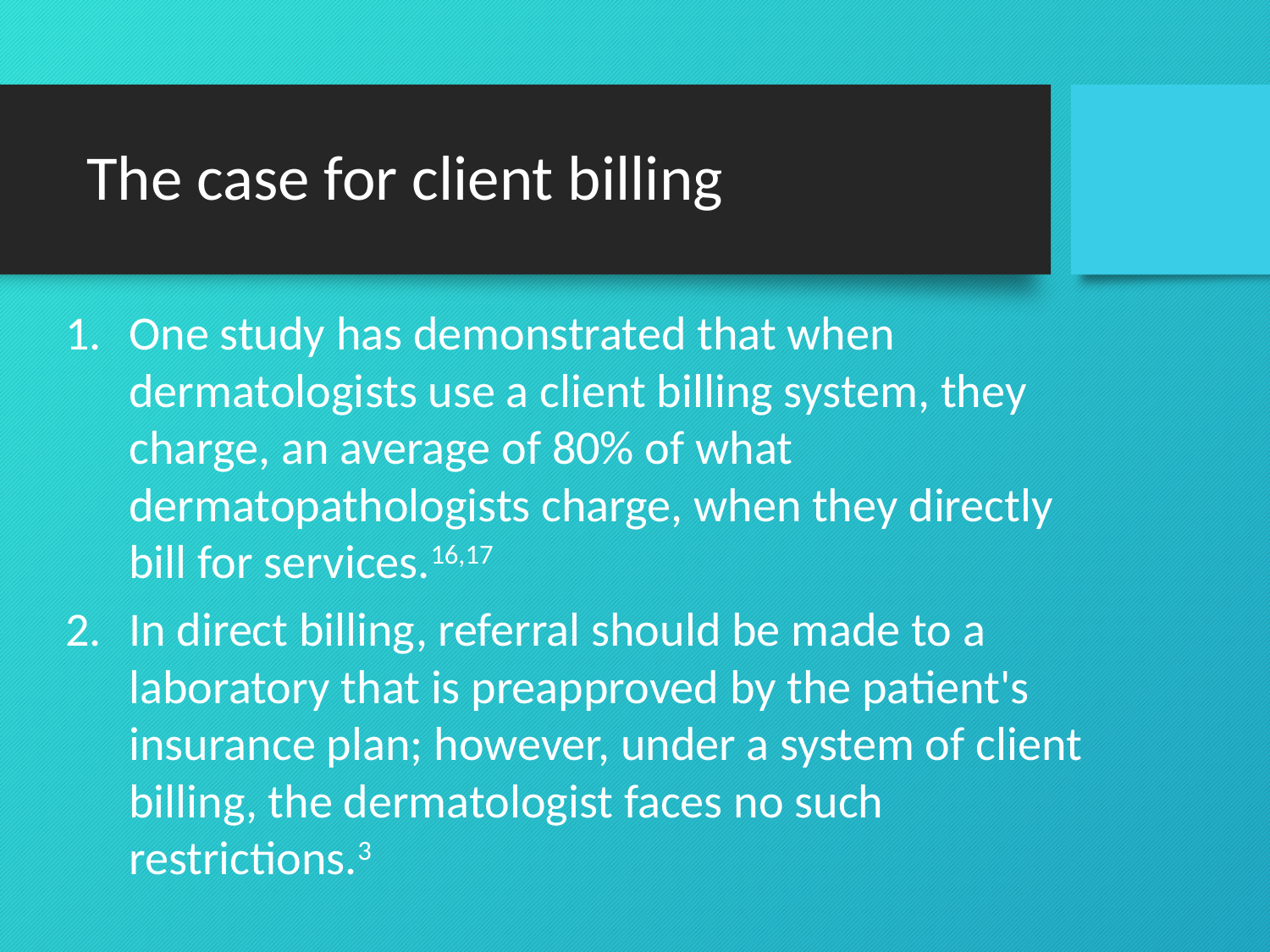

# The case for client billing
One study has demonstrated that when dermatologists use a client billing system, they charge, an average of 80% of what dermatopathologists charge, when they directly bill for services.16,17
In direct billing, referral should be made to a laboratory that is preapproved by the patient's insurance plan; however, under a system of client billing, the dermatologist faces no such restrictions.3

## Slide 29
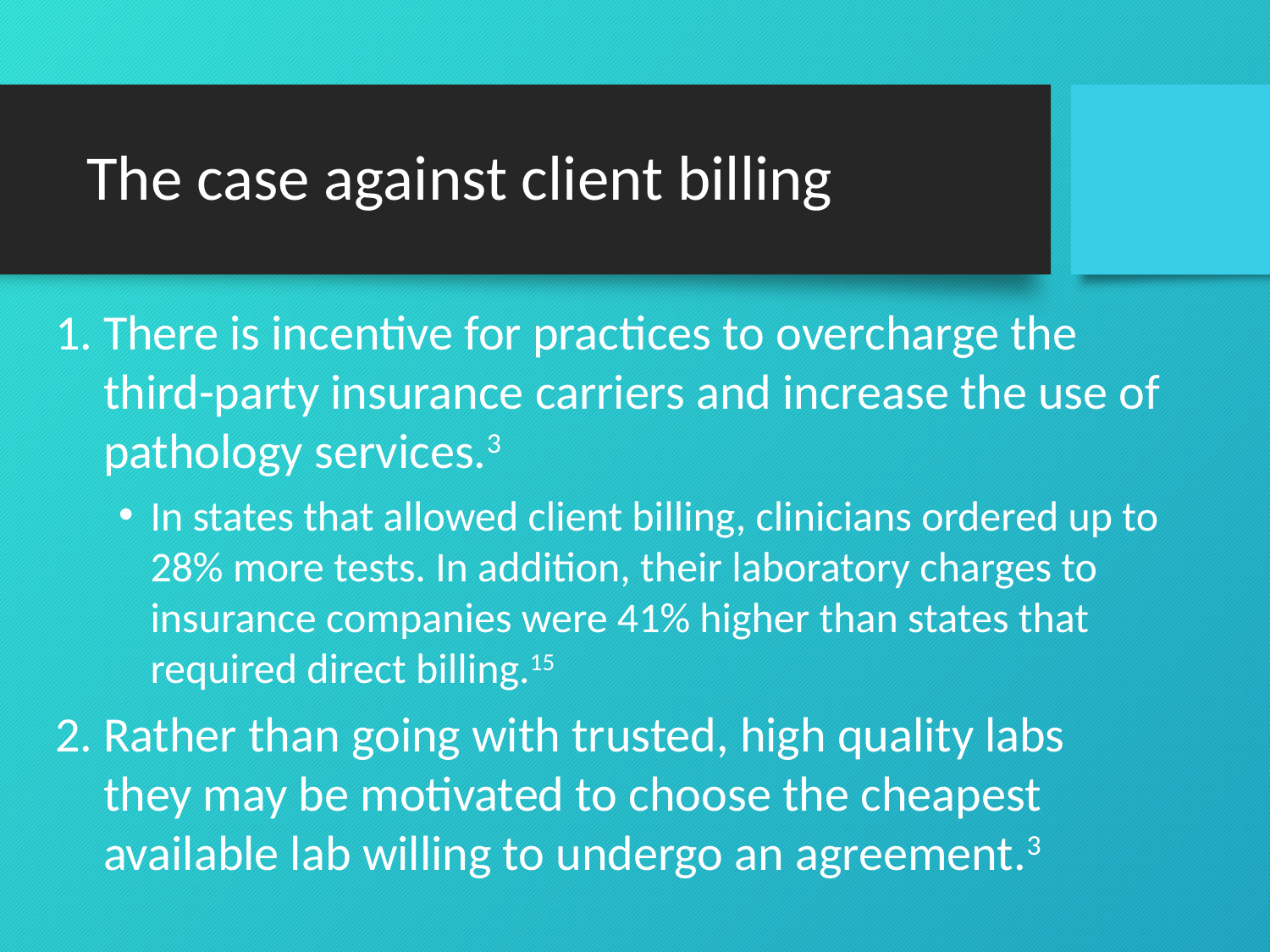

# The case against client billing
There is incentive for practices to overcharge the third-party insurance carriers and increase the use of pathology services.3
In states that allowed client billing, clinicians ordered up to 28% more tests. In addition, their laboratory charges to insurance companies were 41% higher than states that required direct billing.15
Rather than going with trusted, high quality labs they may be motivated to choose the cheapest available lab willing to undergo an agreement.3

## Slide 30
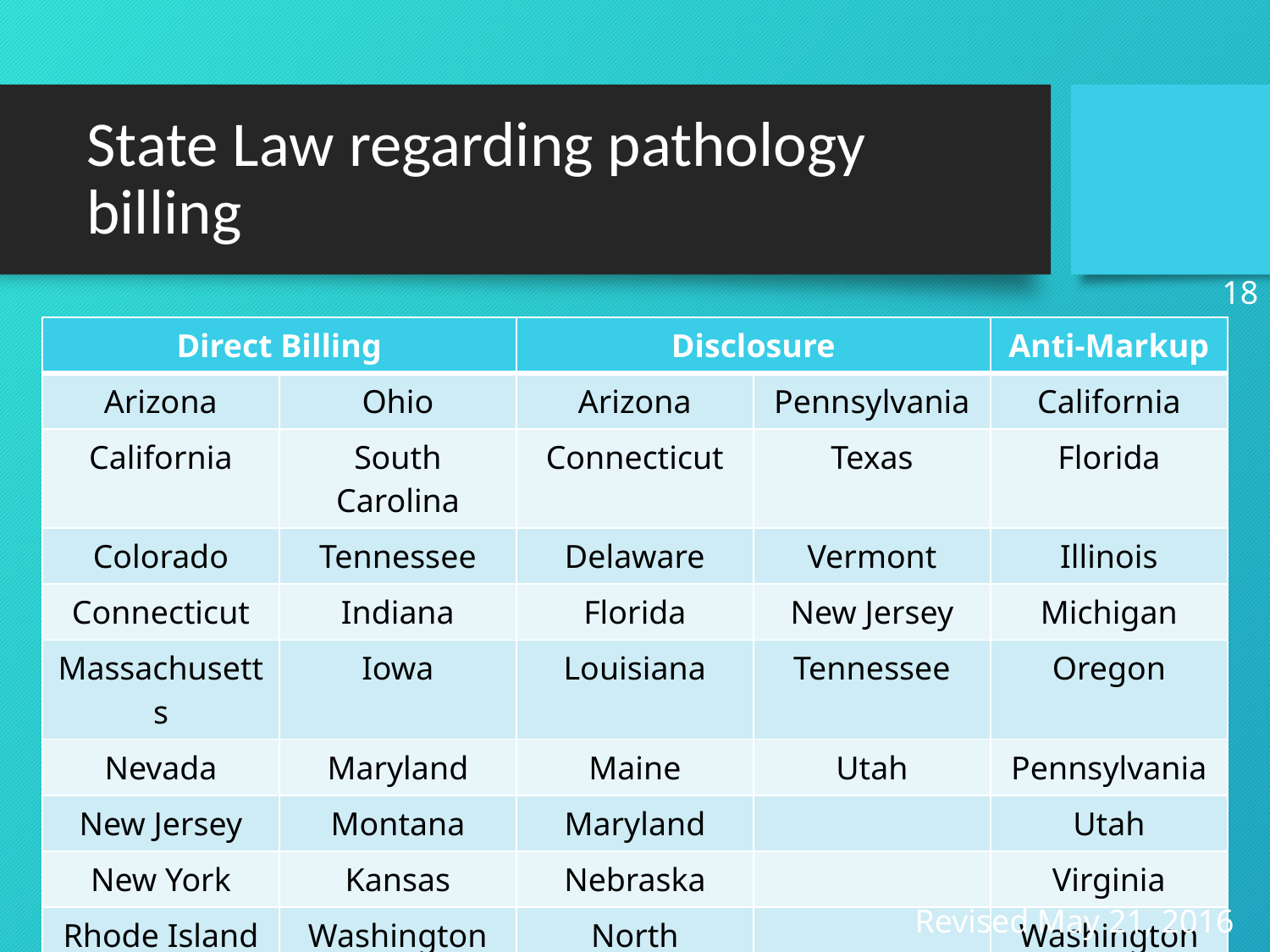

# State Law regarding pathology billing
18
| Direct Billing | | Disclosure | | Anti-Markup |
| --- | --- | --- | --- | --- |
| Arizona | Ohio | Arizona | Pennsylvania | California |
| California | South Carolina | Connecticut | Texas | Florida |
| Colorado | Tennessee | Delaware | Vermont | Illinois |
| Connecticut | Indiana | Florida | New Jersey | Michigan |
| Massachusetts | Iowa | Louisiana | Tennessee | Oregon |
| Nevada | Maryland | Maine | Utah | Pennsylvania |
| New Jersey | Montana | Maryland | | Utah |
| New York | Kansas | Nebraska | | Virginia |
| Rhode Island | Washington | North Carolina | | Washington |
| Louisiana | | Ohio | | |
Revised May 21, 2016

## Slide 31
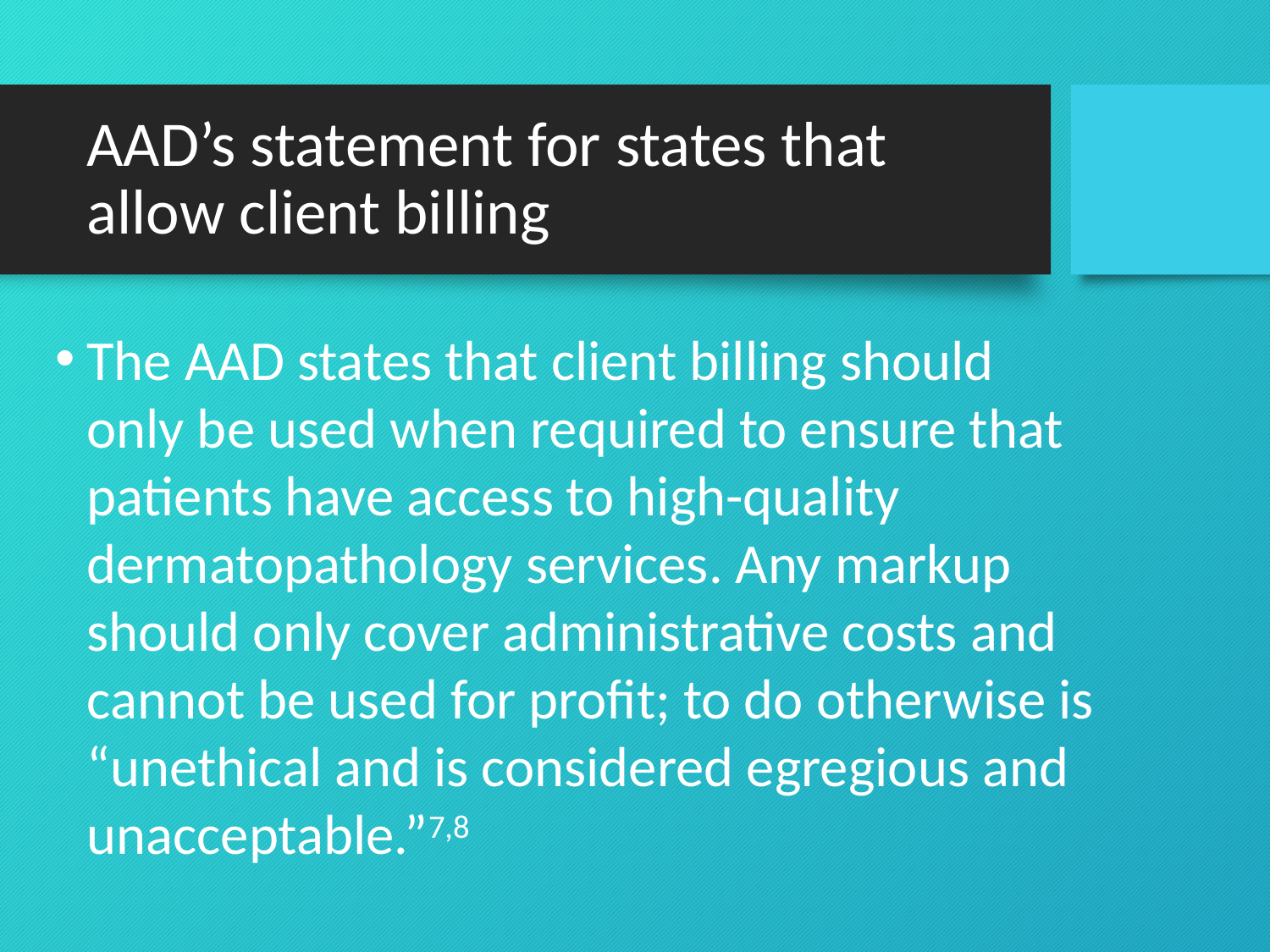

# AAD’s statement for states that allow client billing
The AAD states that client billing should only be used when required to ensure that patients have access to high-quality dermatopathology services. Any markup should only cover administrative costs and cannot be used for profit; to do otherwise is “unethical and is considered egregious and unacceptable.”7,8

## Slide 32
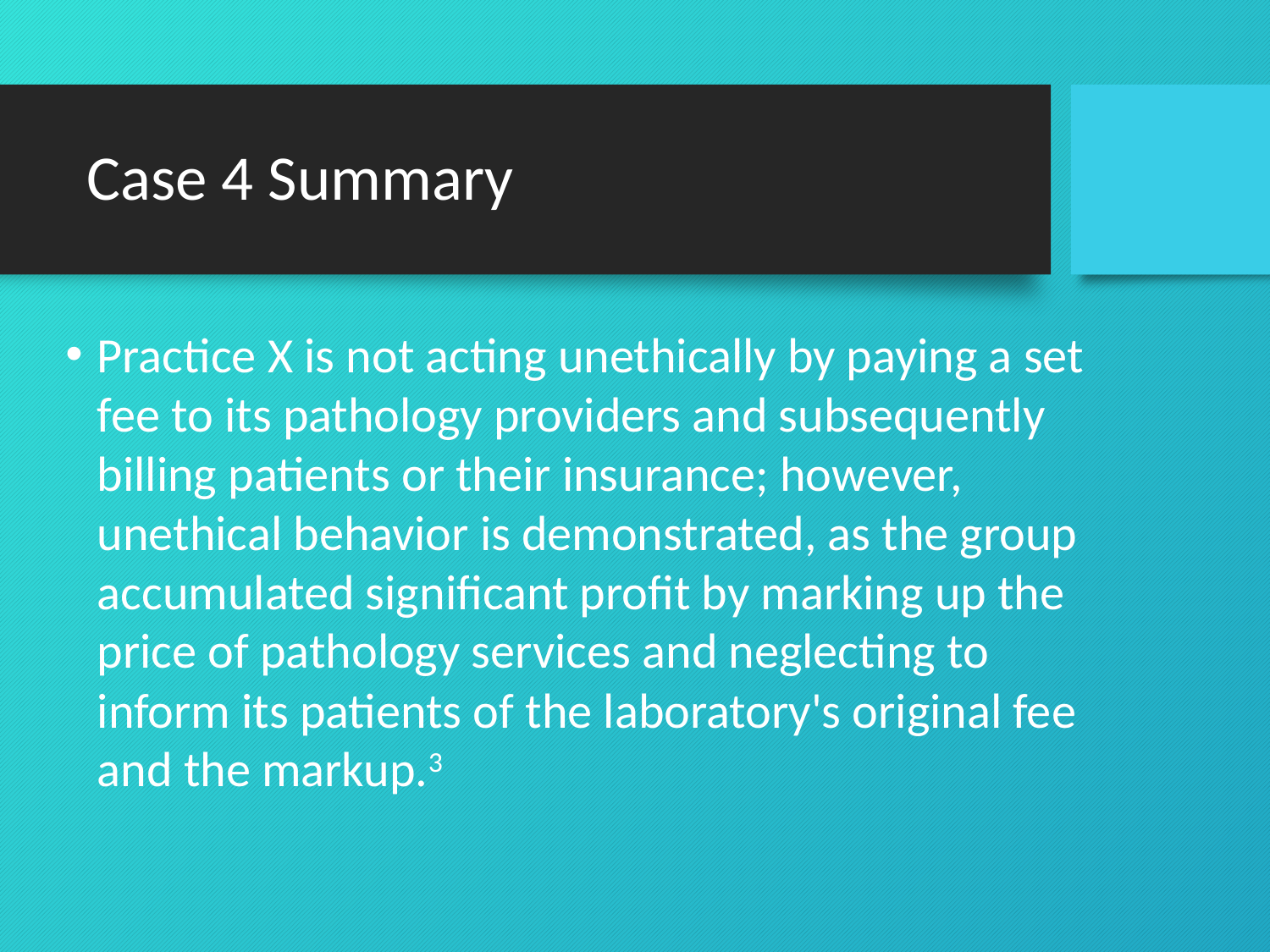

# Case 4 Summary
Practice X is not acting unethically by paying a set fee to its pathology providers and subsequently billing patients or their insurance; however, unethical behavior is demonstrated, as the group accumulated significant profit by marking up the price of pathology services and neglecting to inform its patients of the laboratory's original fee and the markup.3

## Slide 33
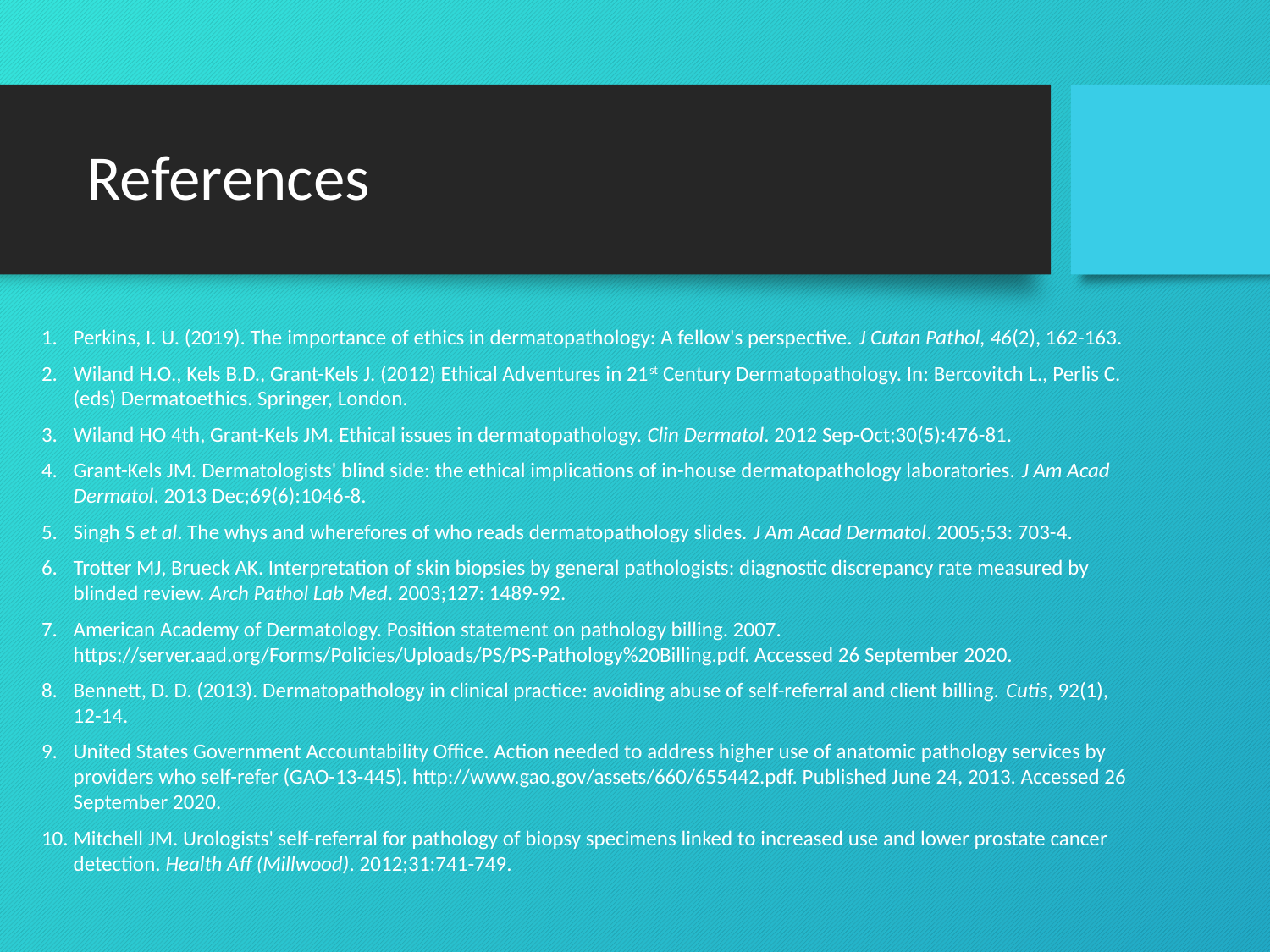

# References
Perkins, I. U. (2019). The importance of ethics in dermatopathology: A fellow's perspective. J Cutan Pathol, 46(2), 162-163.
Wiland H.O., Kels B.D., Grant-Kels J. (2012) Ethical Adventures in 21st Century Dermatopathology. In: Bercovitch L., Perlis C. (eds) Dermatoethics. Springer, London.
Wiland HO 4th, Grant-Kels JM. Ethical issues in dermatopathology. Clin Dermatol. 2012 Sep-Oct;30(5):476-81.
Grant-Kels JM. Dermatologists' blind side: the ethical implications of in-house dermatopathology laboratories. J Am Acad Dermatol. 2013 Dec;69(6):1046-8.
Singh S et al. The whys and wherefores of who reads dermatopathology slides. J Am Acad Dermatol. 2005;53: 703-4.
Trotter MJ, Brueck AK. Interpretation of skin biopsies by general pathologists: diagnostic discrepancy rate measured by blinded review. Arch Pathol Lab Med. 2003;127: 1489-92.
American Academy of Dermatology. Position statement on pathology billing. 2007. https://server.aad.org/Forms/Policies/Uploads/PS/PS-Pathology%20Billing.pdf. Accessed 26 September 2020.
Bennett, D. D. (2013). Dermatopathology in clinical practice: avoiding abuse of self-referral and client billing. Cutis, 92(1), 12-14.
United States Government Accountability Office. Action needed to address higher use of anatomic pathology services by providers who self-refer (GAO-13-445). http://www.gao.gov/assets/660/655442.pdf. Published June 24, 2013. Accessed 26 September 2020.
Mitchell JM. Urologists' self-referral for pathology of biopsy specimens linked to increased use and lower prostate cancer detection. Health Aff (Millwood). 2012;31:741-749.

## Slide 34
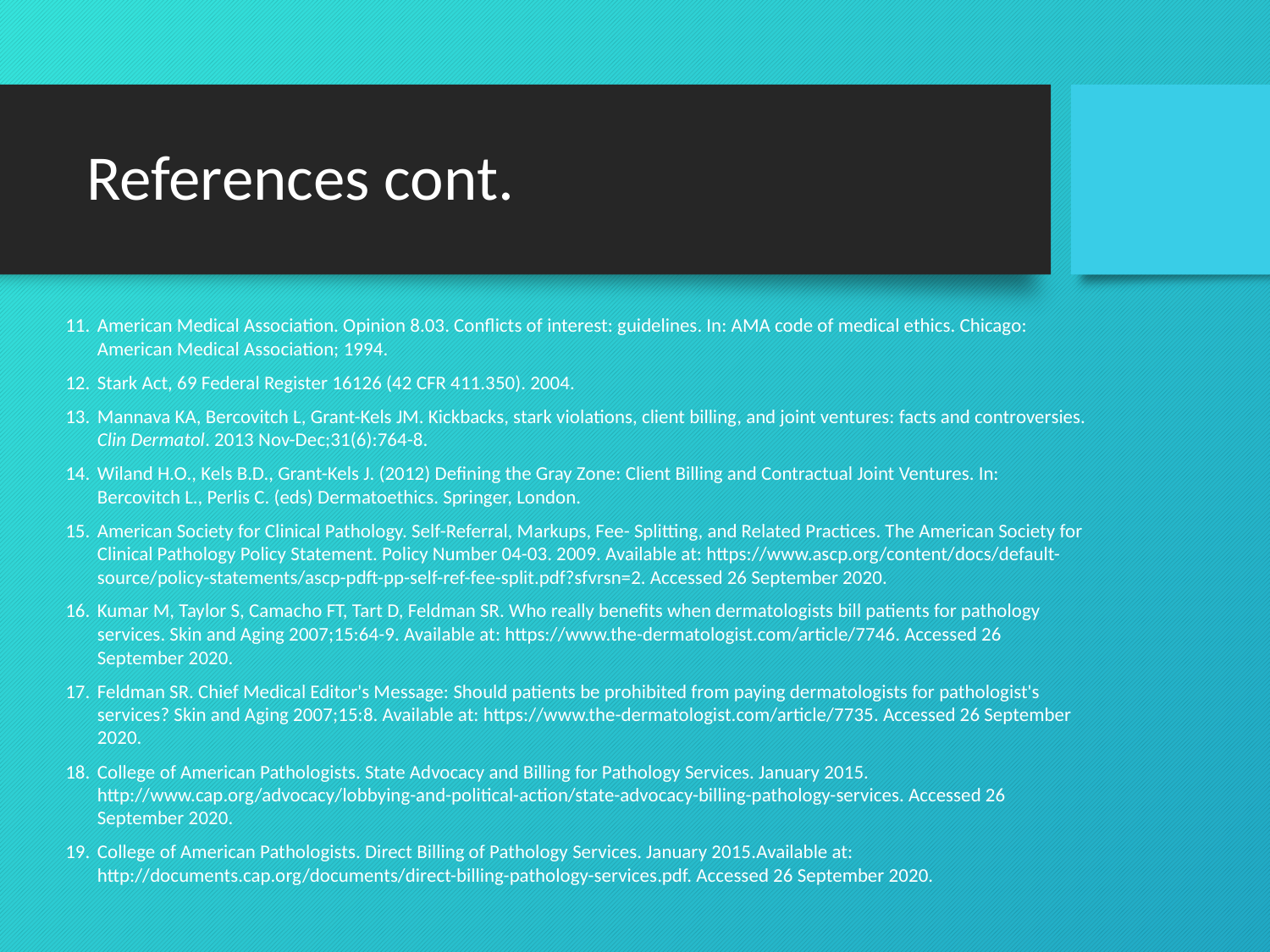

# References cont.
American Medical Association. Opinion 8.03. Conflicts of interest: guidelines. In: AMA code of medical ethics. Chicago: American Medical Association; 1994.
Stark Act, 69 Federal Register 16126 (42 CFR 411.350). 2004.
Mannava KA, Bercovitch L, Grant-Kels JM. Kickbacks, stark violations, client billing, and joint ventures: facts and controversies. Clin Dermatol. 2013 Nov-Dec;31(6):764-8.
Wiland H.O., Kels B.D., Grant-Kels J. (2012) Defining the Gray Zone: Client Billing and Contractual Joint Ventures. In: Bercovitch L., Perlis C. (eds) Dermatoethics. Springer, London.
American Society for Clinical Pathology. Self-Referral, Markups, Fee- Splitting, and Related Practices. The American Society for Clinical Pathology Policy Statement. Policy Number 04-03. 2009. Available at: https://www.ascp.org/content/docs/default-source/policy-statements/ascp-pdft-pp-self-ref-fee-split.pdf?sfvrsn=2. Accessed 26 September 2020.
Kumar M, Taylor S, Camacho FT, Tart D, Feldman SR. Who really benefits when dermatologists bill patients for pathology services. Skin and Aging 2007;15:64-9. Available at: https://www.the-dermatologist.com/article/7746. Accessed 26 September 2020.
Feldman SR. Chief Medical Editor's Message: Should patients be prohibited from paying dermatologists for pathologist's services? Skin and Aging 2007;15:8. Available at: https://www.the-dermatologist.com/article/7735. Accessed 26 September 2020.
College of American Pathologists. State Advocacy and Billing for Pathology Services. January 2015. http://www.cap.org/advocacy/lobbying-and-political-action/state-advocacy-billing-pathology-services. Accessed 26 September 2020.
College of American Pathologists. Direct Billing of Pathology Services. January 2015.Available at: http://documents.cap.org/documents/direct-billing-pathology-services.pdf. Accessed 26 September 2020.
