# Supplementary material for: Structured Case-Based Ethics Discussion for Trainees and Faculty on Dermatopathology
Source: MedEdPORTAL. 2023 May 16;19:11314. doi: 10.15766/mep_2374-8265.11314 (PMC10185701; doi:10.15766/mep_2374-8265.11314)
Supplement: Supplementary file 1 — Dermatoethics Primer.pptxEthics in Dermatopathology.pptxFacilitators Guide.docxFeedback Survey.docx [file mep_2374-8265.11314-s001.zip › C. Facilitators Guide.docx]

Facilitator’s guide for “Dermatoethics in Dermatopathology”

1. Facilitators
   1. Selection of facilitators
      1. In our sessions, facilitators consisted of a dermatology attending, a dermatology resident, and a medical student. We did not require that the facilitators have an extensive background in ethics. However, we did require facilitators to study “Appendix A – Primer in dermatoethics” as well as “Appendix B – Ethics in dermatopathology.”
   2. Assignment of cases
      1. We had 2-3 designated facilitators for each session. Prior to the day of the session, we assigned the four cases in Appendix B evenly among the facilitators. This allowed facilitators to study and prepare to present only the cases they were assigned. If this was the first ethics workshop for the audience, we also assigned the presentation of Appendix A to one of the facilitators.
2. Conduction of the session
   1. Virtual conduction of the session
      1. We conducted the session over the Zoom videoconference platform. A link was provided to participants to join the videoconference. At the time of the session, one of the facilitators was assigned to open the PowerPoint presentation on their screen and share their screen with the audience. This individual advanced the slides at the request of the other facilitators, and took the responsibility of typing audience responses on the screen when a question was posed to the group. When typing audience responses, the facilitator driving the PowerPoint could either use the “annotate” feature of Zoom to type answers directly on the screen, or they could transition to the “edit” mode in PowerPoint to begin typing bullet points on the slide. The remaining facilitators had the PowerPoint open on their individual computers in order to have the presenter notes available for reference. We did not divide the audience into sub-groups, but rather presented the session to the entire group and encouraged the participation of the audience.
   2. In-person conduction of the session
      1. For groups conducting the session in person, we recommend having a projector available to project the PowerPoint presentations. If a whiteboard is available, we recommend using the whiteboard to write down participants’ responses to the questions posed in the session. This may require another facilitator or assistant to run the PowerPoint while another individual writes on the board. Alternatively, one facilitator could type the answers of the audience on the PowerPoint.
3. Order of presentation
   1. Appendix A
      1. We recommend beginning the session with a brief introduction of all the facilitators. If this is the first ethics session for the audience, we recommend starting with the presentation of “Appendix A – Primer in dermatoethics.” This will introduce the audience to the four key principles of ethics, and allow them to use these principles during the discussion in Appendix B. After completing Appendix A, we recommend transitioning to Appendix B.
   2. Appendix B
      1. If the audience has previously had an ethics session, we recommend starting with “Appendix B – Ethics in dermatopathology.” Proceed through the slides in presenter view, starting with an overview of the session objectives, and then transitioning into the discussion cases. For each case, read the case scenario and then invite the audience to begin the discussion with the question posed at the bottom of the case scenario. The speaker’s notes at the bottom of the slide will provide talking points and instructions for the facilitator to help guide the discussion. At times, additional questions or talking points will be provided in the speaker’s notes only, and will not be presented on the slide. Thus, it is important for facilitators to familiarize themselves with the speaker’s notes of their assigned case prior to facilitating the session, as well as to have these available during the actual session. When a question is posed, the speaker’s notes will clarify if there is a space on the PowerPoint presentation to annotate participants’ answers to the question.
