# Supplementary material for: Structured Case-Based Ethics Discussion for Trainees and Faculty on Dermatopathology
Source: MedEdPORTAL. 2023 May 16;19:11314. doi: 10.15766/mep_2374-8265.11314 (PMC10185701; doi:10.15766/mep_2374-8265.11314)
Supplement: Supplementary file 1 — Dermatoethics Primer.pptxEthics in Dermatopathology.pptxFacilitators Guide.docxFeedback Survey.docx [file mep_2374-8265.11314-s001.zip › D. Feedback Survey.docx]

1. Please indicate your level of training:

- Dermatology Attending
- Dermatology Resident
- Medical Student
- Other (please describe)

Objectives:

By the end of this session, participants should be able to:

1. Identify ethical and professionalism issues embedded in our day-to-day practice
2. Make an ethical argument using ethical principles
3. Manage ethical conflicts using appropriate resources
4. Which of the following best describes your reaction to the session?
   - I learned little or nothing
   - I learned a few things
   - I learned a great deal
5. What changes will you incorporate into your future endeavors as a result of the knowledge/perspectives acquired during this activity?
6. For each of the objectives listed below, please check the box under the number that indicated your level of achievement of the objective both **before** and **after** completing the dermatoethics forum
7. No achievement
8. Low achievement
9. Moderate achievement
10. High achievement

|  | BEFORE the forum | | | | AFTER the forum | | | |
| --- | --- | --- | --- | --- | --- | --- | --- | --- |
|  | 1 | 2 | 3 | 4 | 1 | 2 | 3 | 4 |
| Identify ethical and professionalism issues embedded in our day-to-day practice of dermatopathology |  |  |  |  |  |  |  |  |
| Use the four key principles of bioethics to make an ethical argument |  |  |  |  |  |  |  |  |
| Manage ethical conflicts in dermatopathology using appropriate resources |  |  |  |  |  |  |  |  |

1. What was your FAVORITE part of the session?
2. What was your LEAST FAVORITE part of the session?
3. How can we improve the session?
4. Would you recommend this session to a colleague?
5. What future dermatoethics topics would you like addressed?
